# First complete genome sequences and transcript expression profiling of *tea plant necrotic ring blotch virus* isolates from Iran

**DOI:** 10.1371/journal.pone.0354634

**Published:** 2026-08-25

**Authors:** Fereshteh Esmaeilzadeh, Davoud Koolivand, Abozar Ghorbani

**Affiliations:** 1 Department of Plant Protection, Faculty of Agriculture, University of Zanjan, Zanjan, Iran; 2 Nuclear Agriculture Research School, Nuclear Science and Technology Research Institute (NSTRI), Karaj, Iran; Griffith University, AUSTRALIA

## Abstract

*Tea plant necrotic ring blotch virus* (TPNRBV), a highly prevalent and damaging pathogen in tea plantations. In 2021, TPNRBV was detected in Iran, where its molecular and biological properties were subsequently characterized. Despite the growing recognition of TPNRBV, there is a notable lack of complete genome sequences for this virus. In the recent research, High-throughput sequencing (HTS) was performed using the Illumina HiSeq 2000 platform. In silico analyses, including genome assembly, differential expression of TPNRBV open reading frames (ORFs), identification of intra-population single-nucleotide variants (SNVs), as well as phylogenetic and recombination analyses, were performed on the HTS data using CLC Genomics Workbench. The complete genome sequences of two Iranian TPNRBV isolates were obtained using HTS. Analysis of the transcript expression levels for TPNRBV ORFs revealed a significant increase in the expression of the P22 gene, followed by the P24 and P14 genes. Polymorphism analysis identified 93 single-nucleotide polymorphisms (SNVs) across the TPNRBV genome, which were highly concentrated in the RNA1 and RNA2 segments. Phylogenetic analysis based on RNA1 to RNA4 revealed that the Iranian TPNRBV isolates share a close evolutionary relationship with Chinese isolates. In this study, the complete genome sequences of two TPNRBV isolates from Iran are reported for the first time. While the function of P22 remains unclear, its elevated expression in both samples suggests the need for further investigation into its potential role. The identification of amino acid-changing SNVs in the coding regions highlights their evolutionary significance, underscoring the need for further research to understand the impact of these mutations on the virus’s life cycle and pathogenicity. Given that TPNRBV SNVs were identified from pooled samples, a high degree of sequence variation and abundant SNVs were anticipated. This study represents the first comprehensive investigation of tea plant virome in Iran and provides important insights into this field. Additionally, it marks the first report on the population-level expression profile of TPNRBV viral transcripts and the prediction of SNVs, offering a foundation for future research focused on the control and management of this virus.

## Background

The tea plant is an important perennial evergreen woody plant that is extensively cultivated in many Asian, African, and South American countries. The young leaves of the plant are processed to prepare tea (are picked to produce tea), the second most popular beverage in the world after water.

Tea plant necrotic ring blotch disease is a highly damaging condition that significantly affects tea plants across several Asian countries. The disease is caused by tea plant necrotic ring blotch virus (TPNRBV), species *Blunervirus camelliae*, a member of the genus *Blunervirus* in the *Kitaviridae* family, first identified in China in 2018 through metagenomic sequencing [1]. Since its discovery, TPNRBV has been reported in various tea-producing regions, including Iran, Japan, and Indonesia [2–5]. TPNRBV has emerged as one of the most prevalent viruses infecting tea plants in China, alongside Tea plant line pattern virus (TPLPV) and several other viruses identified through metagenomic analyses.

The most noticeable symptoms of TPNRBV infection include necrotic ring blotches on mature leaves, typically in the lower canopy, leading to premature leaf drop and reduced production of new leaves [1]. Additional symptoms, such as leaf discoloration and developmental abnormalities, may lead to plant death. The virus is mainly transmitted through mechanical means and seeds [4]. It is also hypothesized that insect vectors such as mites, aphids, leafhoppers, and whiteflies may contribute to the spread of TPNRBV [6]. This hypothesis is informed by the confirmed role of eriophyid mites (e.g., *Brevipalpus* spp.) as vectors for other viruses within the family Kitaviridae, to which TPNRBV belongs [7,8]. However, to date, no specific insect or mite vector has been identified for TPNRBV. TPNRBV has a restricted host range, primarily infecting tea (*Camellia sinensis*) and *Camellia japonica* [1,9,10].

TPNRBV is an approximately 85 nm spherical virus with a quadripartite, positive-sense, single-stranded RNA genome, where each segment contains a poly (A) tail at its 3′ end. The genome consists of four RNA segments: RNA1, RNA2, RNA3, and RNA4. RNA1 and RNA2 are 5922 and 4107 nucleotides (nt) in length, respectively, and each contains a single open reading frame (ORF). RNA1 encodes a methyltransferase-helicase, while RNA2 encodes a helicase-RNA polymerase. RNA3, 2705 nt long, contains four ORFs. ORF1 (p14) encodes a 372nt protein with an unknown function and shows no significant matches in BLASTp searches. ORF2 (p29) encodes a 753nt protein that shares 94% identity with a hypothetical protein from the *Blueberry necrotic ring blotch virus* (BNRBV). ORF3 (p24), downstream of p29, encodes a 588 nt protein with 98.53% identity to a virion membrane protein from RNA3 of BNRBV and 30.32% identity to a putative virion protein from the Tomato fruit blotch virus. ORF4 (p22), located at the 3′-end of the segment, encodes a 633 nt protein that shows 96.19% identity to a hypothetical protein from BNRBV. RNA4 is 2272 nt in length and contains a single ORF that encodes the movement protein.

TPNRBV has a genome of 14,979 nucleotides, which is larger than the genomes of most plant viruses. Currently, only a limited number of complete genome sequences for TPNRBV are available in GenBank, primarily from isolates in China and Japan [1,3,11],. Sequencing the complete genomes of TPNRBV isolates from different regions can provide valuable insights into the virus’s biology and evolutionary history. High Throughput Sequencing (HTS)-based approaches not only allow for the identification of both known and novel plant-infecting viruses but also enable viral genome assembly, providing a more detailed picture of viral population genetics. HTS techniques also facilitate the detection of single-nucleotide variation (SNVs) without requiring prior knowledge of the host or pathogen [12].

Despite these advancements in genome assembly using HTS, the complete genomes of many viruses, including TPNRBV, remain unavailable in Iran. Furthermore, while TPNRBV is of significant concern, the genetic characteristics of replicative viral populations at the population level, as well as the RNA expression profiles of TPNRBV populations, have yet to be thoroughly analyzed. Equally important for developing control strategies is a deeper understanding of the host plant’s immune landscape during infection [13].

In this study, the complete genome of two TPNRBV isolates was sequenced using HTS. Additionally, we determined the profile of differential gene expression of TPNRBV and identified the occurrence of SNVs in tea transcriptomic data.

This study builds directly upon our previous work [4], which reported the first molecular and biological characterization of TPNRBV in Iran. While the prior study confirmed the virus’s presence and basic biological properties, the current research employs high-throughput sequencing to provide the first complete genome sequences from Iran, enabling advanced genomic, phylogenetic, and population-level expression analyses. Together, these studies offer a comprehensive profile of TPNRBV in the region.

## Materials and methods

### Sample collection

A total of twenty tea leaf samples exhibiting viral disease symptoms were collected in 2021 and 2022 from different tea plantations in the Guilan and Mazandaran provinces in the north of Iran. The collected plants showed a range of virus-like symptoms, including necrotic ring blotch, discoloration (albino and chlorine), yellowing, ring spots, and mosaic. This study is based on integrated analyses of two original transcriptomic datasets generated by our group [14]. The first dataset was produced from an RNA-seq experiment conducted in 2021, and the second from a separate RNA-seq experiment conducted in 2022. The methodology for library preparation and sequencing was consistent across both projects and is detailed in the Materials and Methods section below.

### Total RNA extraction, library construction and sequencing

High-throughput sequencing (HTS) was performed on 20 tea leaf samples. Total RNA was extracted from approximately 100 mg of leaf tissue from each sample using the RiboEX Kit (GeneAll, South Korea) according to the manufacturer’s instructions. The quality and concentration of the extracted RNA were assessed using agarose gel electrophoresis and Nanodrop spectrophotometer (Thermo Fisher Scientific, USA). Equal amounts of RNA from each sample were pooled. Integrity and purity of the total RNA were also analyzed using a Bioanalyzer 2100 based on an RIN number > 7. RNA-Seq libraries were then prepared using the TruSeq Stranded Total RNA Preparation Kit with Ribo-Zero depletion according to the manufacturer’s instructions. The libraries were sequenced by Novogene (China) on an Illumina HiSeq 2000 platform, generating 150 bp paired-end reads.

### Bioinformatic analysis

Raw reads produced from the HTS were processed using CLC Genomics Workbench (version 20, QIAGEN, Venlo, The Netherlands) to remove adapter sequences and low-quality sequences with more than two ambiguous nucleotides. The cleaned and trimmed reads were then mapped to the tea genome (*Camellia sinensis* genome GCF_004153795.1_AHAU_CSS) using CLC Genomics Workbench 20 with default parameters and a length fraction and similarity fraction set to 0.8. From the clean reads, 27,244,799 and 78,269,958 reads were mapped to the tea genome. After removing host-derived reads, the remaining unmapped reads were de novo assembled into contigs using the ‘De Novo Assembly’ tool in CLC Genomics Workbench v20. The assembly was performed with a word size (for read overlap) of 20 and a minimum contig length of 200 nucleotides. The resulting contigs were compared with sequences available in the NCBI viral reference database (https://www.ncbi.nlm.nih.gov/genome/viruses/) using the BLASTn tool within CLC Genomics Workbench. The search parameters were set with an E-value cutoff of 1e-5. For enhanced confidence in virus detection, viral hits were filtered using the following criteria: contig length ≥ 700 nt and Open Blast Output results. Contigs for which the Greatest HSP length in the BLAST results was ≥ 700 nucleotides were considered highly likely to be of viral origin. The nucleotide identity of these selected contigs to their closest viral matches was high (e.g., > 84% for TPNRBV and >78% for CoAV1 isolates identified in this study). This length filter was applied to prioritize the assembly and analysis of conventional plant viruses.

To ensure our analysis was also sensitive to very small viral agents such as viroids, we conducted an additional, BLASTn search of all contigs against the viroid database. No significant hits to small agents (e.g., viroids) were identified in this dataset.

### Verification of HTS data

To confirm the presence of TPNRBV in the individual samples used for the HTS pool, total RNA was reverse transcribed using the Easy cDNA Synthesis Kit (Parstous, Iran) with random hexamer primers, according to the manufacturer’s protocol. TPNRBV presence was confirmed by PCR using two specific primer pairs, TPNRBV3-F/TPNRBV3-R (5’-TTCGCCACTCACAAAGACAACAAACT-3’/5’-GTAGCGGAGCGGAAAGAAAAGACT-3’) and TPNRBV-MP-F/TPNRBV-MP-R (5’- GCGGATCCATGTCTATAGC-3’/5’- ACAAGCTTAAAAGTAATAGGTGGTACAGC −3’) [1,4]. PCR amplifications were performed in a final volume of 25 μL, containing 3 μL of template cDNA, 1.5 μL of each primer, 12.5 μL of 2 × PCR Master Mix, and deionized water to a final volume of 25 μL. The PCR program conditions were as follows: initial denaturation at 95 °C for 5 min, followed by 35 cycles at 95 °C for 30 s, 54 (TPNRBV3-F/R) and 50 (TPNRBV-MP-F/) for 30 s, and 72 °C for 40 s (for the RNA3 segment) or 60 s (for the MP), with a final extension at 72°C for 7 min. The amplified PCR products were electrophoresed in a 1% agarose gel stained with SYBR Safe (SinaClon, Iran) to verify the appropriate fragment size. The obtained amplicons were then subjected to Sanger sequencing by Sinuhe Biotech Company (Iran).

### De novo assembly of the TPNRBV genome and SNV prediction

To assemble the complete genome sequences of TPNRBV from the RNA-Seq data, an initial sensitive mapping step was implemented. Given the low abundance of viral RNA within the host transcriptome and to accommodate potential genetic divergence of the local isolate, permissive alignment parameters were used to maximize viral read recovery. Specifically, trimmed reads were mapped to the TPNRBV reference sequences (NC_040401-NC_040404) using CLC Genomics Workbench 20 with a length fraction and similarity fraction set to 0.5 and 0.8, respectively. To identify SNVs in the assembled virus genomes, the raw RNA-Seq data were mapped to the reference genomes using CLC Genomics Workbench 20 with the following parameters: minimum coverage of 2, minimum variant frequency of 0.01, and maximum variant p-value of 10^−6^. After filtering out synonymous SNVs, the frequency and distribution of polymorphisms across the four segments of the TPNRBV genome were analyzed. SNV prediction was performed by detecting nucleotides that differed from the reference sequences. The positions of identified single-nucleotide variations (SNVs) were visualized on the 3D protein structures by integrating the Protein Data Bank (PDB) from the RCSB PDB database with CLC Genomics Workbench. It is important to note that, as the RNA-seq data originated from a pool of 20 plants, the identified SNVs and their frequencies represent a composite of viral sequences from the entire sampled population. Therefore, a low-frequency variant could represent either a true minor variant within a single host’s viral population or a fixed variant in a virus infecting only a small subset of the plants in the pool.

### TPNRBV gene expression using RNA‑Seq data

Analysis of transcript expression levels for each coding DNA sequence (CDS) in the TPNRBV genome (TPNRBV ORFs) was performed by mapping the trimmed reads to the TPNRBV Refseq using CLC Genomics Workbench with the following parameters: length fraction of 0.8, similarity fraction of 0.8, costs of 2 for mismatches and deletions, and 3 for insertions. The mapping results were used to calculate the transcripts per kilobase million (TPM) for each ORF. TPM normalization was performed using only the total number of reads mapped to the TPNRBV reference genome, thereby enabling comparison of relative expression levels among viral ORFs independent of host-derived reads

### Phylogenetic analysis/ construction of phylogenetic trees

The consensus sequences obtained from the preceding analyses (for the four segments of the TPNRBV genome) were subjected to a BLASTn search to identify the most similar sequences, which were then used to construct phylogenetic trees. The phylogenetic relationships among the Iranian TPNRBV isolate and genome sequences retrieved from GenBank were analyzed using MEGA version 11 [15]. To determine the phylogenetic position of the Iranian TPNRBV isolates, four phylogenetic trees were constructed using the maximum likelihood (ML) method with 1000 bootstrap replicates. The nucleotide substitution models TN93 + G + I, GTR + G, HKY + G, and HKY + G were applied to RNA1-RNA4. Additionally, the nucleotide sequence identity percentages for the four segments of the TPNRBV genome were calculated using the Sequence Demarcation Tool (SDT v1.2) software [16], which was used to generate pairwise nucleotide sequence identity matrices.

### Recombination analysis

Potential recombination events were identified in the four segments of the TPNRBV genome. The analysis was conducted using the Recombination Detection Program (RDP v4.97) [17] with multiple algorithms, including GENECONV, Bootscan, Chimaera, MaxChi, SiScan, 3Seq, and RDP, all run with default settings. Only recombination events detected by at least four of these algorithms were considered reliable and included in the analysis.

## Results

### Collection of tea leaves for tea virome study and library preparation

**Fig 1:**
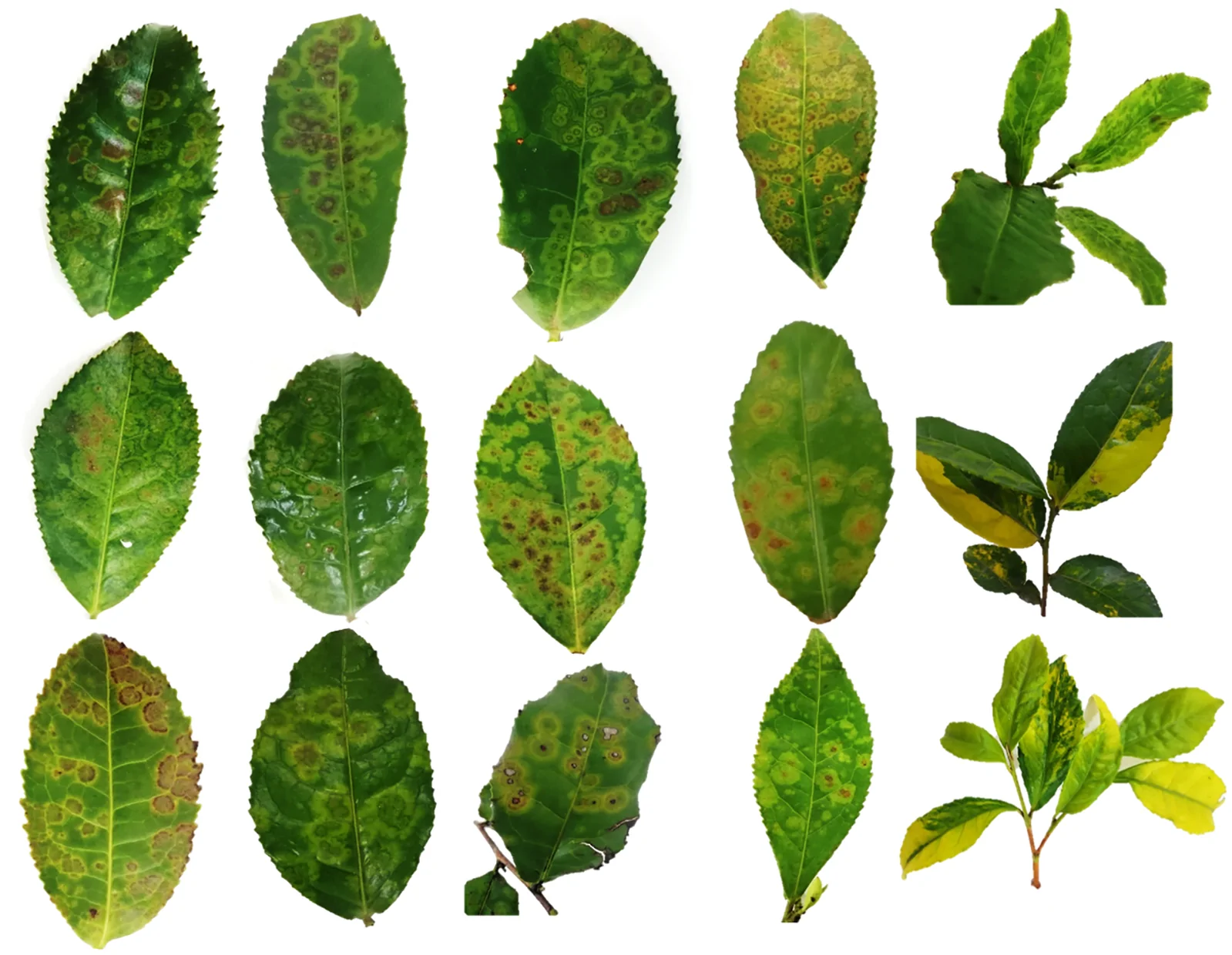
Viral symptoms were observed on tea plants in the tea plantations of Guilan and Mazandaran provinces.

A total of 20 leaf samples were collected from 16 different geographical locations in 2021 and 2022. As shown in Fig 1, the collected tea leaves exhibited symptoms of ring blotch and foliage discoloration (Fig 1).

Total RNAs were extracted from each plant sample, and two composite (pooled) samples were subjected to RNA-Seq. The samples were pooled based on the collection years. Two separate libraries were prepared, and paired-end sequencing was performed using an Illumina HiSeq 2000 system.

### De novo transcriptome assembly

High-throughput sequencing of the two libraries yielded a total of 38,125,708 and 103,830,264 raw paired-end reads for the 2021 and 2022 samples, respectively. After removing adapter sequences, low-quality reads, and host-derived reads, 10,861,237 and 25,603,934 clean reads were obtained from each library, respectively. *De novo* transcriptome assembly of the unmapped reads resulted in 11,638 contigs for library 2021 and 34,454 contigs for library 2022. The approximately threefold increase in the total number of contigs assembled from the 2022 library compared to the 2021 library (34,454 vs. 11,638) is consistent with the proportional increase in clean sequencing data available for assembly (~2.7-fold higher), reflecting expected technical variations between sequencing runs. The number of TPNRBV-specific contigs was similar in both libraries (36 vs. 35), confirming the consistent and dominant presence of the virus in the sampled pools. BLASTn analysis showed that the majority of the contigs were associated with TPNRBV in both libraries. The remaining non-TPNRBV contigs were examined using stringent criteria (contig length ≥ 700 nucleotides and meaningful coverage of viral genomic regions in BLASTn). While some showed short, partial homology to other plant viruses or to non-plant viral sequences (e.g., phages or mycoviruses likely associated with the plant microbiome), none met the thresholds for confident identification of an additional plant virus. Therefore, TPNRBV was confirmed as the dominant and robustly detected plant virus in these transcriptome libraries.

**Fig 2:**
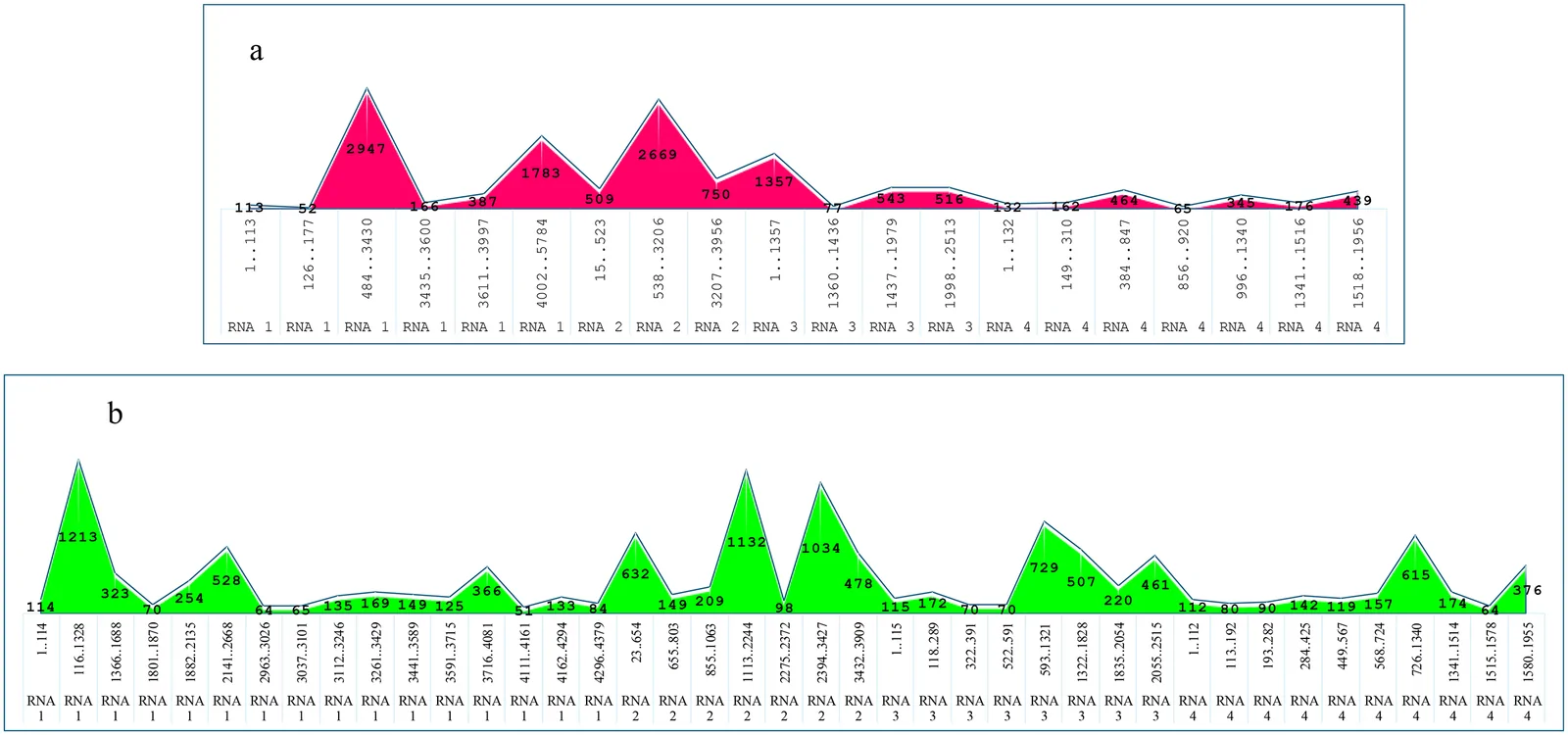
**a, b.** Reads coverage based on alignment of sequenced reads on the reference TPNRBV genome (RNA-seq reads mapping to the reference TPNRBV genome).

A total of 35 TPNRBV-related contigs were identified from library 2021, and 36 from library 2022. Despite the larger total dataset in 2022, the key difference was in the sequencing depth supporting the viral genomes, which was 4- to 5-fold higher (e.g., ~ 3.8 million vs. ~ 0.8 million mapped reads for RNA1). This increased depth provided greater confidence in the genome assemblies and enhanced sensitivity for variant detection. The mapped reads were sufficient to cover the full viral genome, as confirmed by the high-coverage maps (Fig 2a, **b).** Importantly, no region of the TPNRBV genome in either library showed coverage below the minimum threshold of 2 × , and no systematically low-coverage regions were observed, indicating that the assemblies are unlikely to be affected by coverage-related errors.

The depth of coverage across the four genomic segments was not uniform, a common observation in transcriptomic studies of multipartite RNA viruses. Despite this variation, the coverage maps (Fig 2a, 2b) confirm that the entire length of all segments was covered, with no regions falling below the minimum coverage threshold of 2X required for reliable SNV detection. This ensures the completeness of the genome assemblies and the robustness of the variant analysis

The complete genome sequences of the Iranian TPNRBV isolates (designated TPB-Iran and TPNRBV-Ir) were found to be 5922 nt for RNA1, 4107 nt for RNA2, 2678 nt for RNA3, and 2272 nt for RNA4, with poly-A tails. These sequences have been deposited in GenBank under accession numbers ON475416–ON475419 and PV700595- PV700598. The assembled genome sequences showed the highest pairwise nucleotide identity to isolates from China. BLASTn analysis revealed high-confidence matches (E-value = 0.0, Query Coverage = 100%) with the following percent identities for each segment: RNA1: 96.13% to a Fujian isolate (OQ948458); RNA2: 96.38%; RNA3: 97.27%; and RNA4: 94.89% to Hangzhou isolates (NC_040402-NC_040404). Furthermore, BLASTx analysis confirmed the presence of conserved domains characteristic of TPNRBV, including methyltransferase-helicase (RNA1), helicase-RNA polymerase (RNA2), a virion membrane protein (RNA3-P24), and a movement protein (RNA4). A comprehensive pairwise identity matrix is provided in Fig 5.

**Fig 3:**
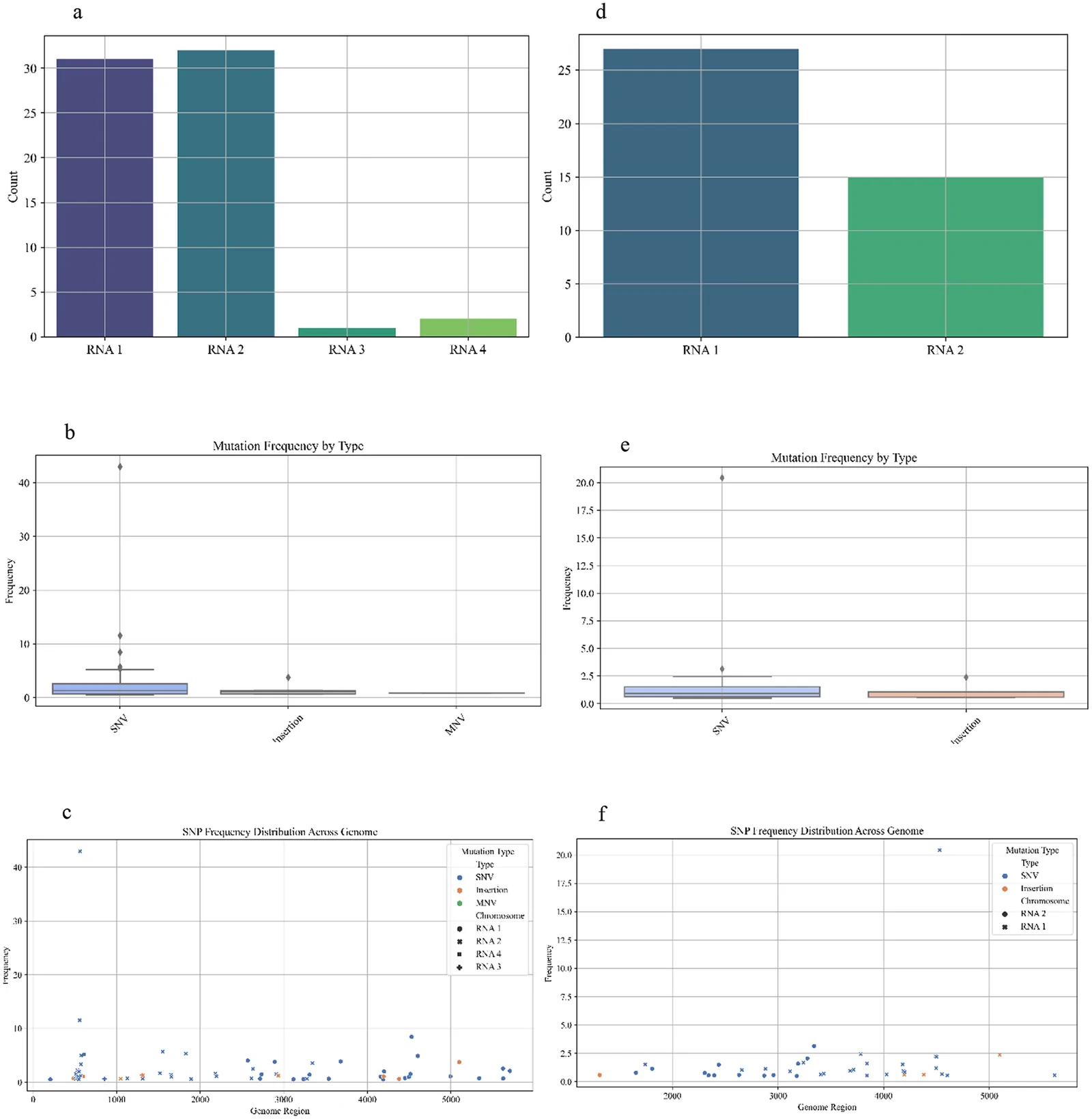
Frequency, type, and positions of identified SNVs on the TPNRBV genome. a, b, c: RNA seq1; d,e,f: RNA seq2.

**Fig 4:**
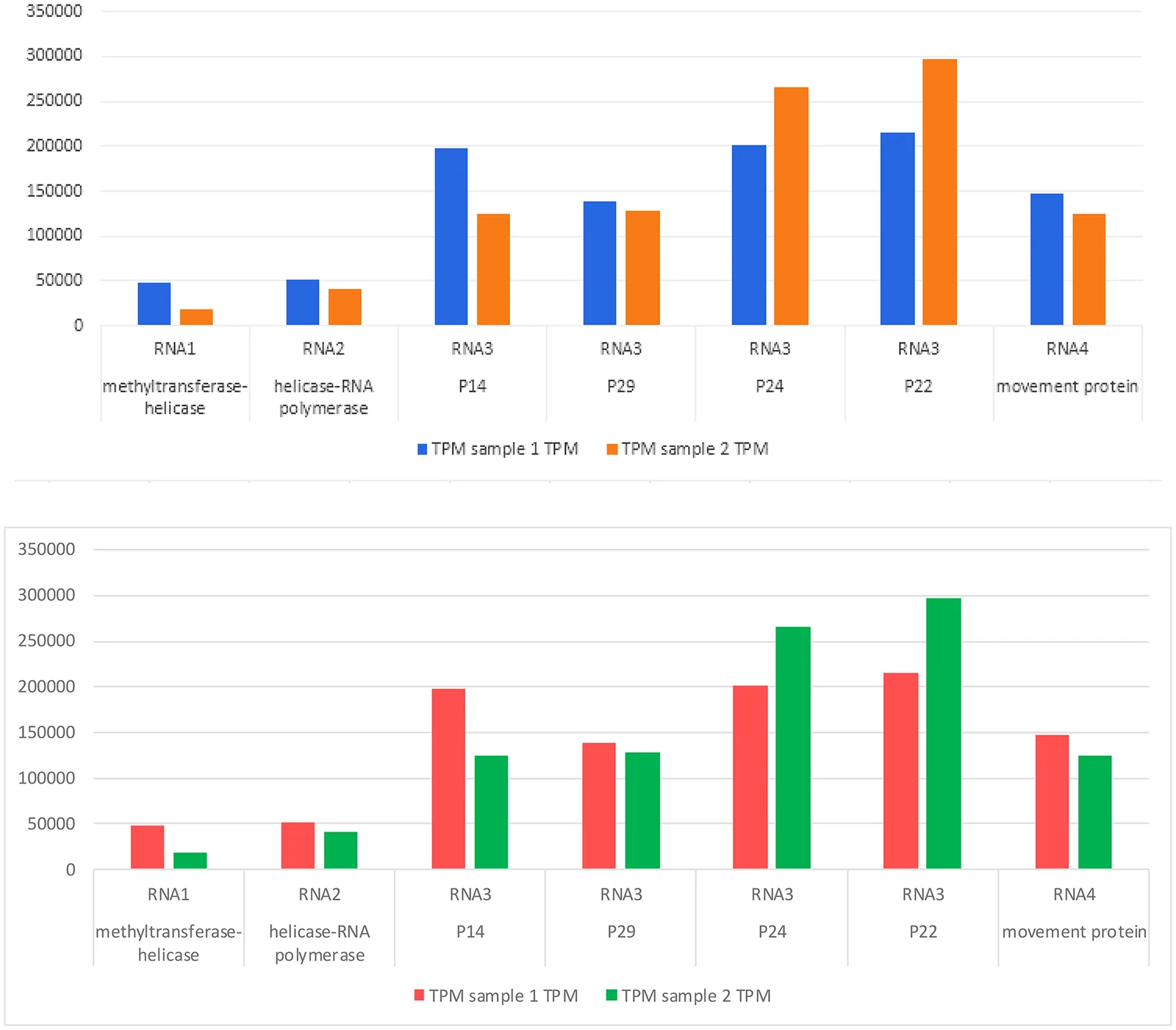
Expression levels (transcript per million, TPM) of Tea plant necrotic ring blotch virus (TPNRBV) in RNA-seq data of infected plants.

**Fig 5:**
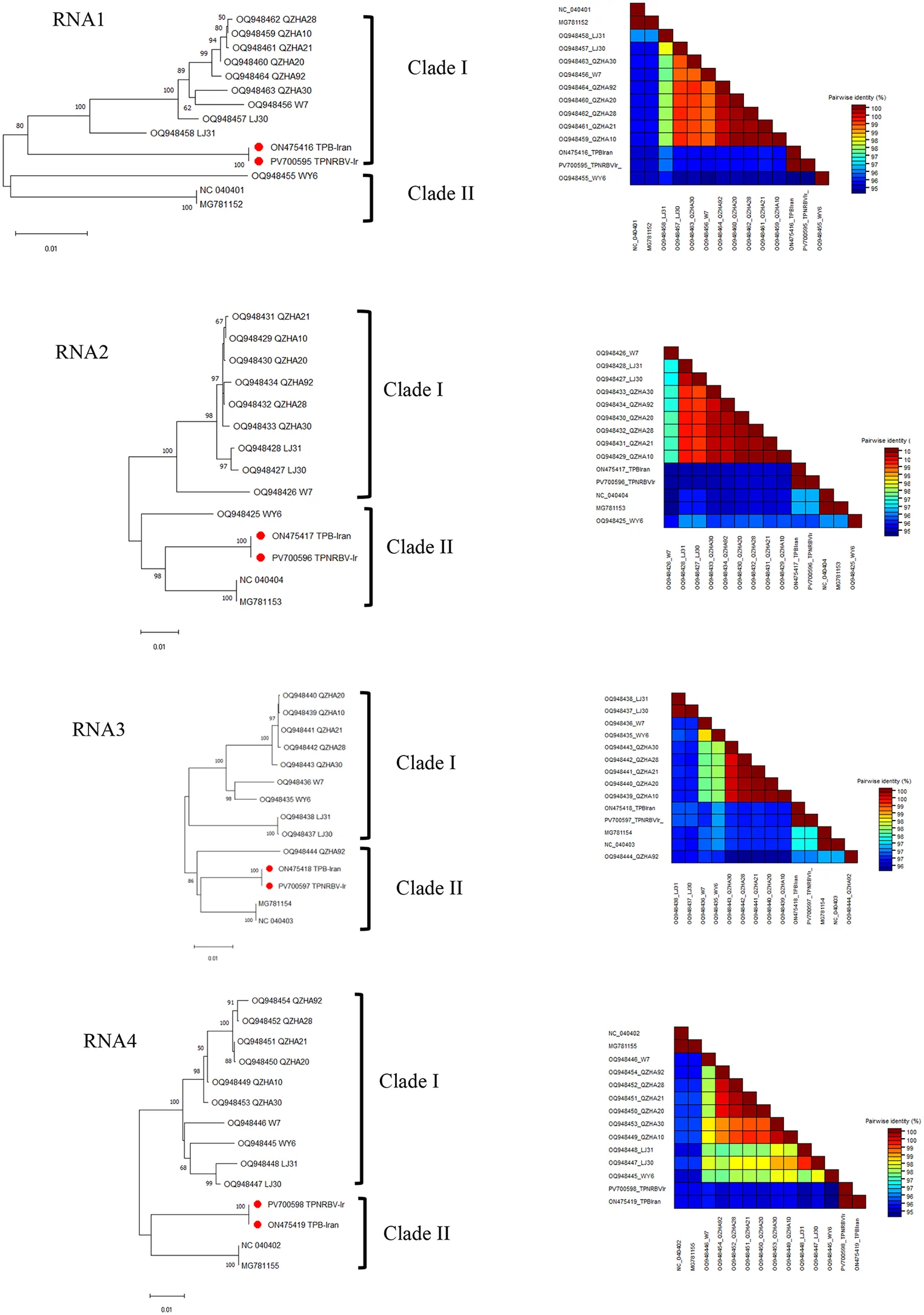
(left) Phylogenetic position of the two Iranian TPNRBV isolates based on the assembled consensus sequences for four segments of TPNRBV. Phylogenetic trees for four TPB-Iran and TPNRBV-Ir segments were constructed using MEGA 11 using the maximum likelihood (ML) method with 1,000 bootstrap replicates. right) Pairwise nucleotide sequence identity matrix was determined using SDT v1.2.

### Identification of sequence variants within the TPNRBV population

To assess the genetic diversity of the TPNRBV population across the sampled tea plants, intra-population single-nucleotide variants (SNVs) were called from the pooled transcriptomic data. By applying a 1% threshold for variant calling, 93 non-synonymous SNVs were identified, with frequencies ranging from 0.26% to 21.47% (Fig 3; S1 Table). The distribution of these frequencies was highly skewed: 90 out of 93 SNVs (96.8%) occurred at frequencies below 5%, consistent with a background of transient mutations. Only three variants were found at frequencies above 5% (5.76%, 14.44%, and 21.47%).

These non-synonymous SNVs were located exclusively in the open reading frame (ORF) regions, with none detected in the untranslated regions (UTRs). The details of the identified SNVs are as follows: 44 SNVs in RNA1, 37 in RNA2, 1 in RNA3, and 1 in RNA4. This uneven distribution resulted in a high density of non-synonymous variants in the replicase genes encoded by RNA1 and RNA2, while the movement protein (RNA4) and most ORFs of RNA3, including the highly expressed P22 gene, showed remarkably low variant counts. The distribution of these 93 non-synonymous SNVs was highly uneven, with the vast majority concentrated in RNA1 and RNA2. In contrast, RNA3 and RNA4 each contained only one SNV, and notably, no SNVs were identified in the P22 gene located in RNA3 at the 1% frequency threshold in this pooled dataset. Furthermore, 15 SNVs were shared between both samples from the 2021 and 2022 collections. The 15 SNVs conserved between the 2021 and 2022 pools are of particular interest, as their persistence may indicate a potential selective advantage or founder effect in the local TPNRBV population; their functional impact merits further investigation. Additionally, 9 insertions and one multi-nucleotide variant (MNV) were found (S1 Table). The distribution of SNVs across the four RNA segments of the TPNRBV genome is shown in Fig 3.

While linking these SNVs to the tertiary structure of the protein using the PDB database, no visible changes were detected in the 3D protein structure. This structural comparison provided a preliminary, qualitative assessment based on visual inspection of the superimposed models. Quantitative analyses of biophysical properties—such as changes in binding pocket volume, electrostatic surface potential, or protein stability—were not performed, as they require specialized molecular dynamics simulations or energy calculations that were beyond the primary genomic focus of this study. It is therefore possible that the identified mutations could affect functional properties not discernible in a static structural alignment.

### Population-level expression profile of TPNRBV ORFs

The relative expression levels of TPNRBV ORFs across the infected plant population were assessed using the pooled RNA-seq data. Comparing the transcripts per million (TPM) values between the two pools, the highest expression was observed for P22, followed by P24, P14, and the movement protein. In contrast, helicase-RNA polymerase and methyltransferase-helicase were expressed at the lowest levels. The expression patterns of TPNRBV ORFs are illustrated in Fig 4. To further assess whether the high expression of the P22 gene could be influenced by technical artifacts, read coverage across the P22 ORF was examined. The P22 region showed continuous read coverage along its entire length in both RNA-seq libraries, with no abrupt drops or localized peaks indicative of assembly errors or post-transcriptional processing. Importantly, coverage across the P22 ORF did not fall below the minimum threshold required for reliable variant detection. The absence of detectable SNVs within P22 further supports the accuracy of its assembly and suggests that its elevated TPM values reflect true high transcript abundance rather than technical bias.

At the population level, our analysis showed no significant difference in viral transcript expression between the two datasets, with P22 being the most abundantly expressed gene and having the highest mRNA levels within the viral transcriptome.

### RT-PCR for validation of the TPNRBV in HTS data

RT-PCR confirmed the presence of TPB-Iran and TPNRBV-Ir in the individual samples used for the pools using two specific primer pairs for TPNRBV. Amplicons of the expected sizes (459 and 948 bp) were obtained and sequenced using Sanger sequencing. BLASTn analysis of the resulting fragments confirmed the presence of TPNRBV in these plants.

RT-PCR analysis of the individual plant RNA extracts that made up the HTS pools confirmed the high prevalence of TPNRBV. The virus was detected in 19 out of 20 while CoAV1 detected in 5 out of the 20 samples (four of them were co-infected with TPNRBV). A single infection of CoAV1 was detected in an individual sample (S2 Table).

### Phylogenetic analysis

The recovered consensus sequences of TPNRBV from the HTS data enabled us to compare the phylogenetic relationships of the TPB-Iran and TPNRBV-Ir isolates with other known TPNRBV isolates. BLASTn searches of the four TPNRBV genome segments showed high nucleotide sequence identity (94.89% to 97.27%) with the TPNRBV isolate from China (TPNRBV_HZ; NC_040401-NC_040404).

To understand the phylogenetic position of the Iranian TPNRBV isolates and gain insights into the virus’s genetic diversity, the complete genome sequence of the TPNRBV isolates obtained in this study were analyzed alongside 12 previously reported TPNRBV isolates. Phylogenetic trees were constructed using the Maximum Likelihood method with 1000 bootstrap replicates in MEGA11. The two major phylogenetic groups in the trees were designated Clade I and Clade II. These clades represent well-supported and consistent divisions, with bootstrap values ≥ 50% at the defining nodes (Fig 5). Detailed information, including accession numbers for the nucleotide sequences used in the phylogenetic analysis, is provided in S3 Table. Since the TPNRBV genome consists of four RNA segments, four independent phylogenetic trees were generated. The analysis of RNA1-RNA2 nucleotide sequences grouped the TPNRBV isolates into two main clades, which were further divided into sub-clades. In the trees constructed from the RNA2, RNA3, and RNA4 sequences, the two Iranian isolates (TPB-Iran and TPNRBV-Ir) were placed in clade II together with isolates from Hangzhou, China (TPNRBV_HZ). However, in the RNA1-based tree, the Iranian isolates were grouped in clade I with those from Fujian (Fig 5).

The high sequence similarity with the TPNRBV isolate from China suggests a potential connection between viral populations in these two geographically distinct locations. Although the phylogenetic tree showed the presence of main clusters, no clear separation by country of origin was observed. Recombination analysis using RDP4 did not detect any recombination events in the analyzed TPNRBV sequences. A pairwise nucleotide sequence identity matrix generated using SDT v1.2 revealed percentage identities of 93.90%–100% for RNA1, 94.10%–100% for RNA2, 95.80%–100% for RNA3, and 95.30%–100% for RNA4 (Fig 5).

The presence or absence of conserved nucleotide motifs at the 5’ termini of TPB-Iran and TPNRBV-Ir genome segments, as described by Maruyama et al. (2022), was also examined. Similar to isolates from Hangzhou, TPB-Iran and TPNRBV-Ir lacked these conserved sequences in RNA2, RNA3, and RNA4. However, such as TPNRBV_HZ, RNA1 of TPB-Iran and TPNRBV-Ir contained the sequence 5’-ATTAACGA-3,’ along with five additional nucleotides before the conserved motif (5’-TGGGGATTACGA-3’).

This shared, specific pattern of motif loss in RNA2-RNA4 coupled with its conserved (and extended) presence in RNA1 constitutes a notable synapomorphy (a derived trait indicative of common ancestry) between the Iranian and Hangzhou isolates. While prior studies suggest such terminal motifs are significant for viral replication and packaging in segmented RNA viruses [11,18], this precise, multi-segment deletion is unlikely to have arisen independently. Therefore, this structural signature provides stronger evidence for a direct evolutionary link between these isolates than nucleotide identity percentages alone.

Consistent with this link, both isolates also share a unique 110-nt insertion in the 3′ UTR of RNA4. In a study by Chen et al. (2023), the variability in the length of the 3’ non-coding regions of TPNRBV RNA4 was attributed to extensive deletions and insertions. A 110-nucleotide insertion, previously identified only in TPNRBV_HZ by Chen et al., was also found in both TPB-Iran and TPNRBV-Ir isolates. This insertion suggests that these isolates may have resulted from a single gene recombination/insertion event.

Considering the high sequence similarity, the congruent reassortment pattern in both Iranian isolates, and their shared unique 110-nt insertion, a marker unlikely to arise independently, the most parsimonious hypothesis for the emergence of TPNRBV in Iran is a single introduction event. This likely involved infected plant material carrying a virus population with mixed ancestry (or a pre-existing reassortant) from China, followed by its local establishment and spread. The evidence does not strongly support a scenario of multiple, independent introductions of different genomic segments.

## Discussion

Building upon our prior HTS-based virome study that identified two viruses (TPNRBV and CoAV1) in tea transcriptomes [14], this study performs a comprehensive genomic analysis specifically on TPNRBV. Here, we focus on the in-depth characterization of TPNRBV by conducting complete genome assembly, single-nucleotide variant (SNV) discovery across its genome, and phylogenetic analysis. These analyses confirm TPNRBV as the dominant virus in the samples and provide novel insights into its population structure and evolution. The presence of CoAV1, first reported by us in tea plants, is noted as part of the initial virome but is not the subject of further genomic analysis in this work.

The complete genome sequences of TPNRBV were assembled from the tea transcriptome by mapping raw reads to reference TPNRBV genomes. Two complete genome sequences of TPNRBV, comprising four segments with a total length of 14,979 nucleotides, were obtained. In addition to virus identification, the HTS data enabled various downstream analyses, including SNV prediction, gene expression profiling, phylogenetic analysis, and recombination studies.

Plant RNA viruses exhibit high mutation rates, leading to genetically diverse populations. Our analysis of pooled samples provided a snapshot of the genetic diversity within the TPNRBV population across the surveyed tea fields. We identified 93 non-synonymous SNVs, indicating the presence of sequence heterogeneity. The highly skewed frequency spectrum (with 96.8% of variants at <5% frequency) is consistent with a background of transient mutations within a viral population. This heterogeneity likely arises from a mixture of closely related isolates infecting different individual plants within the pool, as well as from spontaneous mutations within hosts.

After filtering out synonymous SNVs, 93 non-synonymous SNVs were identified in coding regions across the entire genome, implying potential evolutionary significance. This study utilized RNA-seq data to identify SNVs at an unprecedented scale within the TPNRBV population, providing crucial insights into its genetic diversity. Detection of these SNVs is crucial for assessing the genetic variability of TPNRBV and its potential impact on viral pathogenicity and replication in infected cells. A future study should focus on the distribution and functional significance of the identified variants to better understand their potential impact on viral fitness and pathogenicity. In this study, the distribution of SNV frequencies was heavily biased toward low frequencies, with 90 out of 93 non-synonymous SNVs (96.8%) occurring at frequencies below 5%. Only three variants were found at frequencies above this threshold (5.76%, 14.44%, and 21.47%). This heterogeneous SNV frequency spectrum is a direct consequence of our pooled-sample design, which combines RNA from multiple plants each potentially infected with a distinct TPNRBV isolate. The three higher-frequency outliers may represent variants under different selective pressures or founder effects within the population, warranting further investigation.

In general, plants are often infected by multiple viruses or isolates of a single virus. Since the SNVs in this study were identified from pooled samples, the SNV information represents a collection of multiple isolates rather than a single virus. Although our analysis focused on SNVs from a single assembled virus, the results reflect the presence of diverse isolates due to the pooled nature of the samples.

Similar findings were reported by Jo et al. (2018), who studied the virome of pooled samples and identified high mutation rates for two viruses (*Barley yellow mosaic virus* and *Barley mild mosaic virus*). Their analysis showed significant genetic variation in pooled samples, while individual isolates exhibited low genetic diversity, suggesting that pooled samples may contain a mixture of different isolates or strains [19]. In our study, the low genetic variability observed in the consensus phylogenetic trees is compatible with the circulation of very similar isolates in the field. When pooled, these similar yet distinct isolates would collectively present as a population with numerous low-frequency SNVs, as we observed. Therefore, the most parsimonious explanation for our data is that the pooled samples contained a mixture of very similar, yet distinct, TPNRBV isolates circulating in the plantation, rather than reflecting diverse viral population dynamics within individual hosts. This interpretation aligns with the low genetic variability observed in the consensus phylogenetic trees.

The high density of non-synonymous variants in the replicase genes (RNA1 and RNA2) likely reflects both the inherent genetic plasticity of these regions—often associated with the error-prone RNA-dependent RNA polymerase (RdRp) and other replicase components—and the presence of multiple similar TPNRBV lineages in the surveyed population. While such variation can serve as a substrate for viral adaptation, our pooled-sample design captures population-level diversity across a field. Therefore, the observed pattern is more consistent with the circulation of multiple, closely related lineages than with conclusive evidence for within-host adaptive evolution or the selection of escape mutants in individual plants.

Furthermore, the 15 non-synonymous SNVs conserved between the 2021 and 2022 sampling seasons, all located in replicase genes, represent compelling targets for future studies aiming to elucidate their potential role in local adaptation or viral fitness.

The expression levels of TPNRBV ORFs were also examined using RNA-seq data, with the highest expression observed for P22, followed by P24 and P14. Although the functions of P22 and P14 remain unclear, the notably high expression of P22 suggests it may play a significant role in the infection cycle. In many plant viruses, highly expressed accessory proteins often function as RNA silencing suppressors (VSRs) or facilitate host-virus interactions [20]. Based on its high expression and this established functional analogy, we hypothesize that P22 is a prime candidate for such functions in TPNRBV. This presents a testable hypothesis for future studies. Therefore, further studies are necessary to elucidate the specific function of this gene. As the virus replicates, understanding the role of these proteins is essential for developing targeted control strategies.

*Kitavirus* genomes often contain orphan ORFs (ORFans), which encode proteins with low similarity to those in public databases. The P24 protein, the second most highly expressed gene, encodes a protein that, based on BLASTp analysis, shows significant similarity to the virion membrane protein encoded by RNA3 in BNRBV.

Differential expression analysis is crucial for understanding the pathogenicity of TPNRBV. Based on transcript expression levels, P22, which is highly expressed in the pooled samples, stands out as an ideal candidate for future control strategies targeting TPNRBV. The lack of high-frequency variants in P22 in our population-level data suggests it is a genetically stable region, highlighting its potential as a prime target for developing diagnostic tools for TPNRBV.

In this study, phylogenetic analysis of the assembled consensus sequences for all four genome segments revealed that TPB-Iran and TPNRBV-Ir share a close evolutionary relationship with TPNRBV isolates from China (Hangzhou/Zhejiang). Low genetic variability was observed between TPNRBV isolates based on the four constructed phylogenetic trees, which is consistent with the findings of Chen et al. (2023) that suggest low genetic diversity among TPNRBV isolates. Recombination analysis, which is essential for understanding the role of recombination in the genetic diversity and evolution of TPNRBV, showed no evidence of recombination among the isolates included in this study. Recombination is a crucial mechanism for generating diversity in RNA viruses, and the absence of recombination may explain the observed reduction in genetic diversity in these TPNRBV populations. The limited availability of TPNRBV sequences in public databases may have contributed to this lack of evidence.

However, an intriguing finding of our phylogenetic analysis was the incongruent placement of the Iranian isolates: they clustered with isolates from Fujian province in the RNA1 tree, but with the Hangzhou isolate in the trees for RNA2, RNA3, and RNA4 (Fig 5). Since no evidence of intra-segment recombination was detected using RDP4, this pattern is most parsimoniously explained by genomic reassortment, a common evolutionary mechanism for viruses with multipartite genomes [21]. This suggests that the Iranian TPNRBV isolates could be natural reassortants, possibly originating from a mixed infection of two parental lineages circulating in China—one lineage related to the Fujian isolates (donating RNA1) and another related to the Hangzhou isolate (donating RNAs 2–4). The subsequent long-distance movement of this reassortant strain to Iran could have occurred through the exchange of infected plant material. While convergent evolution or insufficient sequence diversity in public databases could also contribute to such topological discrepancies, reassortment remains the most direct explanation for the observed segment-specific clustering. This hypothesis highlights the potential for reassortment to generate genetic novelty in TPNRBV and underscores the importance of obtaining full-genome sequences from diverse geographical regions to understand the segment exchange dynamics and global spread of this emerging virus. Therefore, more sequence data from other tea-producing regions would allow for a more thorough investigation into the role of recombination and reassortment in the evolution of TPNRBV.

The unique 110-nt insertion identified in the 3′ UTR of RNA4 in both the Iranian and Hangzhou isolates—absent in all other known TPNRBV isolates—likely represents an independent insertion event in their evolutionary history. Since no recombination signal was detected in this region, it may have arisen through replication-associated indel mechanisms common in viral RNA genomes. Although located in a non-coding region, such insertions can influence RNA secondary structure, stability, or replication efficiency. Importantly, its presence does not conflict with the robust phylogenetic signal from RNA1, which confirms the Iranian isolate as a distinct lineage. This insertion serves as a notable molecular marker for tracking the spread or divergence of specific TPNRBV subpopulations

This finding indicates the stability of the TPNRBV genome and the potential impact of recombination on other plant RNA viruses. This study represents the first report on the complete genome characterization of TPNRBV from Iran. Additionally, it is the first time RNA-seq data has been used to identify mutations and compare the expression levels of TPNRBV ORFs in the host plant. Overall, our in-silico analysis using plant transcriptome data provides valuable insights into viral genome assembly, SNVs, phylogenetic relationships, and genetic recombination, offering essential resources for further research on TPNRBV and its management.

### Considerations on the pooled-sample design

The interpretations of this study are framed by its pooled-sample design. While this approach efficiently captures population-level diversity and expression profiles, it has inherent limitations. First, the SNV frequencies reported represent a composite from multiple plants; a variant at 1% frequency could be a rare mutation in one host’s viral population or a fixed difference in a virus infecting 1 out of 20 plants. Consequently, our data describe the genetic structure of the TPNRBV population at the field level but cannot delineate within-host evolutionary dynamics. Second, the TPM values reflect average viral gene expression across all infected tissue in the pool and should not be interpreted as temporal expression changes within a single infection. Furthermore, the SNVs reported here were identified bioinformatically; their independent validation by methods such as Sanger sequencing remains a necessary step for future work. Despite these constraints, our study provides valuable insights into the population genomics and expressed repertoire of TPNRBV in Iranian tea plantations, establishing a foundation for more detailed within-host studies in the future.

### Limitations and future perspectives

A critical next step is the functional prioritization of the identified SNVs. Specifically, the three high-frequency non-synonymous variants (at 5.76%, 14.44%, and 21.47%) represent prime candidates for *in silico* predictive analysis using tools such as SIFT or PROVEAN to assess whether their amino acid changes are deleterious, neutral, or potentially advantageous. Subsequent experimental validation will be crucial to determine their actual impact on viral protein function, fitness, and pathogenicity.

## Supporting information

S1 Table Single nucleotide polymorphisms (SNVs) among a population of RNA-seq reads, TPB-Iran and TPNRBV-Ir in tea. (DOCX)

S2 Table Summary of sample collection and RT-PCR detection results for TPNRBV and CoAV1, aggregated by major geographical region. (DOCX)

S3 Table Information on Tea plant necrotic ring blotch virus sequences used in phylogenetic analysis. (DOCX)
