# Supplementary material for: First complete genome sequences and transcript expression profiling of tea plant necrotic ring blotch virus isolates from Iran
Source: PLoS One. 2026 Aug 25;21(8):e0354634. doi: 10.1371/journal.pone.0354634 (PMC13505901; doi:10.1371/journal.pone.0354634)
Supplement: Supplementary file 1 [file pone.0354634.s001.docx]

Supplementary Table 1. Single nucleotide variants (SNVs) among a population of RNA-seq reads, TPB-Iran and TPNRBV-Ir in tea

| Chromosome | Region | Type | Reference | Allele | Origin tracks | Sample count | Total number of samples | Mean allele frequency | Amino acid change |
| --- | --- | --- | --- | --- | --- | --- | --- | --- | --- |
| RNA 1 | 206 | SNP | C | T | Sample 1 | 1 | 2 | 0.26648 |  |
| RNA 1 | 486^487 | Insertion | - | T | Sample 1 | 1 | 2 | 0.357619 |  |
| RNA 1 | 1305^1306 | Insertion | - | T | Sample 1 | 1 | 2 | 0.671686 |  |
| RNA 1 | 1742 | SNV | A | G | Sample 2 | 1 | 2 | 0.762188 |  |
| RNA 1 | 2569 | SNV | A | G | Sample 1 | 1 | 2 | 2.012176 |  |
| RNA 1 | 2658 | SNV | A | T | Sample 2 | 1 | 2 | 0.514306 |  |
| RNA 1 | 2716 | SNV | A | G | Sample 1 | 1 | 2 | 0.324708 |  |
| RNA 1 | 2734 | SNV | G | A | Sample 1 | 1 | 2 | 0.736434 |  |
| RNA 1 | 2880 | SNV | A | T | Sample 2 | 1 | 2 | 0.561448 |  |
| RNA 1 | 2891 | SNV | A | C | Sample 1 | 1 | 2 | 1.90408 |  |
| RNA 1 | 3115 | SNV | G | T | Sample 1, Sample 2 | 2 | 2 | 0.715592 | YP_009551524.1:p.Val995Phe |
| RNA 1 | 3235 | SNV | G | A | Sample 1 | 1 | 2 | 0.285335 |  |
| RNA 1 | 3241 | SNV | T | A | Sample 2 | 1 | 2 | 0.835694 |  |
| RNA 1 | 3308 | SNV | G | A | Sample1 | 1 | 2 | 0.702099 |  |
| RNA 1 | 3404 | SNV | G | A | Sample 2 | 1 | 2 | 0.321805 |  |
| RNA 1 | 3433 | SNV | A | G | Sample 2 | 1 | 2 | 0.35629 |  |
| RNA 1 | 3538 | SNV | A | G | Sample 1 | 1 | 2 | 0.319231 |  |
| RNA 1 | 3680 | SNV | A | G | Sample 1 | 1 | 2 | 1.9303 |  |
| RNA 1 | 3684 | SNV | C | A | Sample 2 | 1 | 2 | 0.476347 |  |
| RNA 1 | 3715 | SNV | C | T | Sample 2 | 1 | 2 | 0.531068 |  |
| RNA 1 | 3784 | SNV | C | T | Sample 2 | 1 | 2 | 1.221005 |  |
| RNA 1 | 3842 | SNV | A | C | Sample 2 | 1 | 2 | 0.790823 |  |
| RNA 1 | 3842 | SNV | A | G | Sample 2 | 1 | 2 | 0.266335 |  |
| RNA 1 | 4021 | SNV | T | C | Sample 2 | 1 | 2 | 0.31799 |  |
| RNA 1 | 4027 | SNV | A | G | Sample 2 | 1 | 2 | 0.315145 |  |
| RNA 1 | 4156 | SNV | C | A | Sample 1 | 1 | 2 | 0.56143 |  |
| RNA 1 | 4160 | SNV | A | G | Sample 1 | 1 | 2 | 0.520108 |  |
| RNA 1 | 4180 | SNV | T | C | Sample 2 | 1 | 2 | 0.763383 |  |
| RNA 1 | 4186 | SNV | G | A | Sample 2 | 1 | 2 | 0.467815 |  |
| RNA 1 | 4189 | SNV | A | G | Sample 1 | 1 | 2 | 0.261374 |  |
| RNA 1 | 4195^4196 | Insertion | - | A | Sample 1, Sample 2 | 2 | 2 | 0.834138 | YP_009551524.1:p.Ile1357fs |
| RNA 1 | 4201 | SNV | A | G | Sample 1, Sample 2 | 2 | 2 | 1.425791 | YP_009551524.1:p.Ile1357Val |
| RNA 1 | 4379^4380 | Insertion | - | A | Sample 1, Sample 2 | 2 | 2 | 0.615139 | YP_009551524.1:p.Asn1418fs |
| RNA 1 | 4450 | SNV | G | A | Sample 1 | 1 | 2 | 0.386591 |  |
| RNA 1 | 4495 | SNV | G | A | Sample 1, Sample 2 | 2 | 2 | 1.599941 | YP_009551524.1:p.Ala1455Thr |
| RNA 1 | 4496 | SNV | C | T | Sample 1, Sample 2 | 2 | 2 | 1.086043 | YP_009551524.1:p.Ala1455Val |
| RNA 1 | 4498 | SNV | T | C | Sample 2 | 1 | 2 | 1.095133 |  |
| RNA 1 | 4516 | SNV | T | A | Sample 1 | 1 | 2 | 0.761649 |  |
| RNA 1 | 4530 | SNV | G | A | Sample 1, Sample 2 | 2 | 2 | 14.44553 | YP_009551524.1:p.Met1466Ile |
| RNA 1 | 4552 | SNV | G | A | Sample 2 | 1 | 2 | 0.325735 |  |
| RNA 1 | 4603 | SNV | C | T | Sample 1, Sample 2 | 2 | 2 | 2.718843 | YP_009551524.1:p.Leu1491Phe |
| RNA 1 | 4995 | SNV | A | T | Sample 1 | 1 | 2 | 0.541943 |  |
| RNA 1 | 5099^5100 | Insertion | - | T | Sample 1, Sample 2 | 2 | 2 | 3.050896 | YP_009551524.1:p.Leu1658fs |
| RNA 1 | 5339 | SNV | T | C | Sample 1 | 1 | 2 | 0.366606 |  |
| RNA 1 | 5618 | SNV | A | T | Sample 2 | 1 | 2 | 0.276264 |  |
| RNA 1 | 5621 | SNV | C | T | Sample 1 | 1 | 2 | 1.275924 |  |
| RNA 1 | 5623 | SNV | G | T | Sample 1 | 1 | 2 | 1.267848 |  |
| RNA 1 | 5624 | SNV | T | C | Sample 1 | 1 | 2 | 0.352063 |  |
| RNA 1 | 5705 | SNV | T | C | Sample 1 | 1 | 2 | 1.051982 |  |
| RNA 2 | 508 | SNV | A | T | Sample 1 | 1 | 2 | 0.316125 |  |
| RNA 2 | 515 | SNV | G | A | Sample 1 | 1 | 2 | 0.76761 |  |
| RNA 2 | 526..527 | MNV | CA | AC | Sample 1 | 1 | 2 | 0.430052 |  |
| RNA 2 | 531 | SNV | C | A | Sample 1 | 1 | 2 | 0.371635 |  |
| RNA 2 | 531 | SNV | C | T | Sample 1 | 1 | 2 | 1.12041 |  |
| RNA 2 | 538 | SNV | A | T | Sample 1 | 1 | 2 | 0.531449 |  |
| RNA 2 | 547 | SNV | A | G | Sample 1 | 1 | 2 | 0.268266 |  |
| RNA 2 | 548 | SNV | A | T | Sample 1 | 1 | 2 | 0.993539 |  |
| RNA 2 | 558 | SNV | A | G | Sample 1 | 1 | 2 | 5.766081 |  |
| RNA 2 | 561 | SNV | A | G | Sample 1 | 1 | 2 | 21.48908 |  |
| RNA 2 | 572 | SNV | A | C | Sample 1 | 1 | 2 | 0.599166 |  |
| RNA 2 | 572 | SNV | A | T | Sample 1 | 1 | 2 | 1.653352 |  |
| RNA 2 | 576 | SNV | G | A | Sample 1 | 1 | 2 | 2.492407 |  |
| RNA 2 | 1048^1049 | Insertion | - | A | Sample 1 | 1 | 2 | 0.325907 |  |
| RNA 2 | 1128 | SNV | A | G | Sample 1 | 1 | 2 | 0.357512 |  |
| RNA 2 | 1312 | SNV | G | A | Sample 1, Sample 2 | 2 | 2 | 0.645963 | YP_009551530.1:p.Arg398Lys |
| RNA 2 | 1312^1313 | Insertion | - | A | Sample 1, Sample 2 | 2 | 2 | 0.932203 | YP_009551530.1:p.Asn400fs |
| RNA 2 | 1519 | SNV | T | C | Sample 1 | 1 | 2 | 0.84644 |  |
| RNA 2 | 1550 | SNV | C | A | Sample 1 | 1 | 2 | 2.854406 |  |
| RNA 2 | 1650 | SNV | A | T | Sample 1 | 1 | 2 | 0.697267 |  |
| RNA 2 | 1655 | SNV | A | T | Sample 1, Sample 2 | 2 | 2 | 0.898327 | YP_009551530.1:p.Leu512Phe |
| RNA 2 | 1808 | SNV | G | T | Sample 2 | 1 | 2 | 0.571101 |  |
| RNA 2 | 1827 | SNV | G | A | Sample 1 | 1 | 2 | 2.658818 |  |
| RNA 2 | 1892 | SNV | A | C | Sample 1 | 1 | 2 | 0.294256 |  |
| RNA 2 | 1892 | SNV | A | T | Sample 1 | 1 | 2 | 0.312484 |  |
| RNA 2 | 2181 | SNV | T | A | Sample 1 | 1 | 2 | 0.788385 |  |
| RNA 2 | 2193 | SNV | A | T | Sample 1 | 1 | 2 | 0.551824 |  |
| RNA 2 | 2305 | SNV | T | C | Sample 2 | 1 | 2 | 0.378783 |  |
| RNA 2 | 2342 | SNV | A | C | Sample 2 | 1 | 2 | 0.276408 |  |
| RNA 2 | 2396 | SNV | A | T | Sample 2 | 1 | 2 | 0.271296 |  |
| RNA 2 | 2439 | SNV | A | G | Sample 2 | 1 | 2 | 0.746224 |  |
| RNA 2 | 2612 | SNV | T | A | Sample 1 | 1 | 2 | 0.350501 |  |
| RNA 2 | 2631 | SNV | A | G | Sample 1, Sample 2 | 2 | 2 | 1.546781 | YP_009551530.1:p.Lys838Glu |
| RNA 2 | 2869 | SNV | A | T | Sample 2 | 1 | 2 | 0.262288 |  |
| RNA 2 | 2910 | SNV | G | A | Sample 1 | 1 | 2 | 0.770302 |  |
| RNA 2 | 2933^2934 | Insertion | - | T | Sample 1 | 1 | 2 | 0.611831 |  |
| RNA 2 | 2957 | SNV | G | T | Sample 2 | 1 | 2 | 0.285734 |  |
| RNA 2 | 3178 | SNV | T | C | Sample 2 | 1 | 2 | 0.250394 |  |
| RNA 2 | 3189 | SNV | G | A | Sample 2 | 1 | 2 | 0.79203 |  |
| RNA 2 | 3278 | SNV | A | T | Sample 1, Sample 2 | 2 | 2 | 1.345996 | YP_009551530.1:p.Lys1053Asn |
| RNA 2 | 3342 | SNV | A | T | Sample 1, Sample 2 | 2 | 2 | 3.335378 | YP_009551530.1:p.Ser1075Cys |
| RNA 3 | 854 | SNV | T | C | Sample 1 | 1 | 2 | 0.319794 |  |
| RNA 4 | 603^604 | Insertion | - | A | Sample 1 | 1 | 2 | 0.545805 |  |
| RNA 4 | 609 | SNV | A | G | Sample 1 | 1 | 2 | 2.599949 |  |
