# Supplementary material for: First complete genome sequences and transcript expression profiling of tea plant necrotic ring blotch virus isolates from Iran
Source: PLoS One. 2026 Aug 25;21(8):e0354634. doi: 10.1371/journal.pone.0354634 (PMC13505901; doi:10.1371/journal.pone.0354634)
Supplement: Supplementary file 3 [file pone.0354634.s003.docx]

| Isolate Location | Host | Year | GenBank accession number | | | |
| --- | --- | --- | --- | --- | --- | --- |
|  |  |  | RNA1 | RNA2 | RNA3 | RNA4 |
| TPNRBV_HZ China:  Hangzhou: Zhejiang  TPNRBV-WY6 China: Fujian | *Camellia sinensis*  *Camellia sinensis* | 2018  2023 | NC_040401, MG781152  OQ948455 | NC_040404, MG781153  OQ948425 | NC_040403, MG781154    OQ948435 | NC_040402, MG781155  OQ948445 |
| TPNRBV- W2 China: Fujian | *Camellia sinensis* | 2023 | OQ948456 | OQ948426 | OQ948436 | OQ948446 |
| TPNRBV-LJ30 China: Fujian | *Camellia sinensis* | 2023 | OQ948457 | OQ948427 | OQ948437 | OQ948447 |
| TPNRBV-LJ31 China: Fujian | *Camellia sinensis* | 2023 | OQ948458 | OQ948428 | OQ948438 | OQ948448 |
| TPNRBV-QZHA10 China: Fujian | *Camellia sinensis* | 2023 | OQ948459 | OQ948429 | OQ948439 | OQ948449 |
| TPNRBV-QZHA20 China: Fujian | *Camellia sinensis* | 2023 | OQ948460 | OQ948430 | OQ948440 | OQ948450 |
| TPNRBV-QZHA21 China: Fujian | *Camellia sinensis* | 2023 | OQ948461 | OQ948431 | OQ948441 | OQ948451 |
| TPNRBV-QZHA28 China: Fujian | *Camellia sinensis* | 2023 | OQ948462 | OQ948432 | OQ948442 | OQ948452 |
| TPNRBV-QZHA30 China: Fujian | *Camellia sinensis* | 2023 | OQ948463 | OQ948433 | OQ948443 | OQ948453 |
| TPNRBV-QZHA92 China: Fujian | *Camellia sinensis* | 2023 | OQ948464 | OQ948434 | OQ948444 | OQ948454 |

Supplementary Table 3. Information on Tea plant necrotic ring blotch virus sequences used in phylogenetic analysis
