# Supplementary material for: First complete genome sequences and transcript expression profiling of tea plant necrotic ring blotch virus isolates from Iran
Source: PLoS One. 2026 Aug 25;21(8):e0354634. doi: 10.1371/journal.pone.0354634 (PMC13505901; doi:10.1371/journal.pone.0354634)
Supplement: Supplementary file 4 [file pone.0354634.s005.docx]

Dear Editor,

Thank you for your valuable reviewers’ comments. Your feedback has significantly contributed to the enhancement of our manuscript, and we have addressed all the points raised by reviewers 1, 2, and 3. Please don't hesitate to share any additional comments or questions you may have; we are committed to further improving our manuscript. The changes have been meticulously documented using the different color feature in Microsoft Word, and we have provided detailed responses to each of the reviewer's comments.

**Reviewer #1**

*The authors should mention their previous work on this virus from Scientific Reports (2023) and its connection to the current work. I see a considerable thematic similarity with this previously published work. As part of the review process, I do not have access to the supplementary files for the manuscript.*

We thank the reviewer for raising this important point. To clearly establish the connection between the previous and current studies, we have added the following paragraph at the end of the *Background* section:

“This study builds directly upon our previous work (Esmaeilzadeh et al., 2023), which reported the first molecular and biological characterization of TPNRBV in Iran. While that study confirmed the presence of the virus and described its basic biological properties, the present research applies high-throughput sequencing to generate the first complete genome sequences of Iranian TPNRBV isolates. This approach enables comprehensive genomic, phylogenetic, and population-level analyses. Together, these studies provide an integrated and detailed characterization of TPNRBV in Iran.”

This addition clarifies the logical progression from our initial discovery and characterization of TPNRBV to the current in-depth genomic investigation and highlights the novel contributions of the present manuscript.

**Reviewer #1**

*The images are missing numbers*

Thank you for your comment. The figure numbers have now been added to the images as requested

**lines 42:**

*The abstract repeats information about gene expression*

Thank you for your valuable comment. As suggested, the redundant sentence concerning transcript expression analysis has been removed from the Abstract to avoid repetition.

**lines 59-61:**

*Blunervirus camelliae does not infect anything. It is a taxonomic construct. The name of the virus that infects tea plants is tea plant necrotic ring blotch virus.
Therefore, the sentence*

*"The disease is caused by Blunervirus camelliae (tea plant necrotic ring blotch virus (TPNRBV)), a member of the genus Blunervirus in the Kitaviridae family, first identified in China in 2018 through metagenomic sequencing."
should read as follows:*

The sentence was revised according to the reviewer's comment as follows: The disease is caused by tea plant necrotic ring blotch virus (TPNRBV), species Blunervirus camelliae, a member of the genus Blunervirus in the Kitaviridae family, first identified in China in 2018 through metagenomic sequencing

**line 216:**

*RT-PCR does not confirm the presence of the virus in the dataset, but in the plant.*

The sentence was revised according to the reviewer's comment as follows: RT-PCR confirmed the presence of TPB-Iran and TPNRBV-Ir in the individual samples used for the pools using two specific primer pairs for TPNRBV

**line 219:**

*The aim of sequencing RT-PCR products should be to confirm the sequences obtained using HTS, not to compare them with sequences in GenBank.*

The sentence was revised according to the reviewer's comment as follows: BLASTn analysis of the resulting fragments confirmed the presence of TPNRBV in these plants.

**lines 86, 255, 264:**

*change NGS to HTS*

NGS was changed to HTS.

**lines 188-189:**

*similarity to other isolates – please specify and provide a link to images with sequence comparisons*

We thank the reviewer for this suggestion. We have updated the text to specify the similarity. The pairwise nucleotide identity between our Iranian isolates (TPB-Iran/TPNRBV-Ir) and the closest isolates in GenBank is as follows:

- RNA1: **96.13%** identity with the TPNRBV Fujian isolate.
- RNA2: **96.38%** identity with the TPNRBV Hangzhou isolate (NC_040402).
- RNA3: **97.27%** identity with the TPNRBV Hangzhou isolate (NC_040403).
- RNA4: **94.89%** identity with the TPNRBV Hangzhou isolate (NC_040404).

As requested, we provide a visual comparison in the form of a pairwise identity matrix, which is presented in **Fig. 5**. This figure allows for easy comparison of sequence similarity across all isolates analyzed in this study.

**lines 231-234:**

*The description of the phylogenetic relationships of Iranian isolates does not correspond to the figure*

We thank the reviewer for this comment. To eliminate any ambiguity, we have now **added clear labels (Clade I and Clade II) to the relevant branches in Figure 5**. The description in the text (The two major phylogenetic groups in the trees were designated Clade I and Clade II. These clades represent well-supported and consistent divisions, with bootstrap values ≥ 50% at the defining nodes (Fig. 5)) accurately corresponds to the labeled topology in the revised figure

**lines 191 (and 268-271):**

*these are data from pooled samples, not from a single plant. Therefore, I would not use the term quasispecies, which refers to a virus in a single plant.*

 We thank the reviewer for this crucial comment. We fully agree. The term "quasispecies" has been removed throughout the manuscript (including in the Abstract, Results, and Discussion sections) and replaced with more accurate descriptors such as "genetic diversity within the viral population" and "intra-population sequence variation" to reflect that our data derive from pooled samples representing a field population

**Reviewer #2**

**lines 101-102:**

*Beyond necrotic ring blotch and discoloration, what other specific "virus-like symptoms" were observed and recorded during the sample collection?*

Thank you for your question. Other specific symptoms were indeed observed during sample collection. We have clarified this in the 'Sample collection' subsection. The added text is:
"...including necrotic ring blotch, discoloration (albino and chlorine), yellowing, ring spots, and mosaic."

**line 103:**

*The text refers to two pre-existing transcriptomic datasets [10]. Please give two transcriptomic datasets generation and references.*

We thank the reviewer for this suggestion. We have now clarified the origin of the two transcriptomic datasets in the Materials and Methods section. The added text reads: "This study is based on integrated analyses of two original transcriptomic datasets generated by our group. The first dataset was produced from an RNA-seq experiment conducted in 2021, and the second from a separate RNA-seq experiment conducted in 2022. The methodology for library preparation and sequencing was consistent across both projects and is detailed herein.

**line 104:**

*Please add data regarding samples used for RT-PCR of both detected viruses (numbers, locations, symptoms…as well as their results in the Results section)*

We thank the reviewer for highlighting the importance of these validation data. All requested information is included in the table below:

**Table 2. Summary of sample collection and RT-PCR detection results for TPNRBV and CoAV1, aggregated by major geographical region.**

| Region (Province) | No of Samples | Predominant Symptoms | TPNRBV-Positive | CoAV-Positive | Co-infection  (TPNRBV+CoAV1) |
| --- | --- | --- | --- | --- | --- |
| Guilan Province  Mazandaran Province  Total | 12  8  20 | necrotic ring blotch  discoloration (albino and chlorine)  yellowing,  ring spots  mosaic | 11  8  19 | 3  2  5 | 3  1  4 |

We believe these data, presented in the indicated sections, provide a clear and complete account of the sample set and validation results."

**lines 109-110:**

*About the library preparation, please write the type of RNA library which was constructed.*

RNA-Seq libraries were then prepared using the TruSeq Stranded Total RNA kit according to the manufacturer's instructions. The libraries were sequenced by Novogene (China) on an Illumina HiSeq 2000 platform, generating 150 bp paired-end reads.

**lines 110-111:**

*Concerning quality control: Beyond agarose gel electrophoresis, were any more sensitive methods (such as Bioanalyzer or Qubit) used to quantify RNA integrity (RIN) and concentration? Please write the specific quality thresholds for an RNA sample to be included in the pool.*

The quality and concentration of the extracted RNA were assessed using agarose gel electrophoresis and Nanodrop spectrophotometer (Thermo Fisher Scientific, USA). Equal amounts of RNA from each sample were pooled. Integrity and purity of the total RNA were also analyzed using a Bioanalyzer 2100 based on an RIN number > 7. RNA-Seq libraries were then prepared using the TruSeq Stranded Total RNA Preparation Kit with Ribo-Zero depletion according to the manufacturer's instructions. The libraries were sequenced by Novogene (China) on an Illumina HiSeq 2000 platform, generating 150 bp paired-end reads.

**lines 113-116:**

*Regarding the host subtraction step, please, write the specific alignment parameters (e.g., percentage of identity, length fraction) which were used to map the reads to the (Camellia sinensis) genome (GCF_004153795.1), and the percentage of the total cleaned reads which were successfully mapped and removed as host-derived ones.*

The cleaned and trimmed reads were then mapped to the tea genome (Camellia sinensis genome GCF_004153795.1_AHAU_CSS) using CLC Genomics Workbench 20 with default parameters and a length fraction and similarity fraction set to 0.8. From the clean reads, 27,244,799 and 78,269,958 reads were mapped to the tea genome.

**lines 118-119:**

*Please add more details about the BLAST filtering criteria. The section mentions filtering with "Open Blast Output results." What specific E-value and percentage identity thresholds were used for the BLASTn and BLASTx searches to define a significant viral hit? Adding these data would be more informative.*

We thank the reviewer for this insightful comment. To provide full clarity, we have revised the relevant section in the manuscript. Our filtering criteria involved the following steps:

1. Bioinformatics Screening: Contigs with a Greatest HSP Length ≥ 700 nucleotides in the BLASTn results (using an E-value cutoff of 1e-5) were selected as candidates. Their nucleotide identity to known viruses was high (e.g., >84% for TPNRBV).
2. Experimental Verification: The viruses reported in this study were definitively identified and confirmed using virus-specific RT-PCR assays, followed by Sanger sequencing. This verification is detailed in the 'Verification of HTS data' section.

Furthermore, an unrestricted BLASTn search of all contigs was conducted to detect very small agents like viroids, with no significant hits found.

These revisions now clearly reflect our selection criteria and confirmatory path.

**line 118:**

*For the TPNRBV contigs, what were the key BLAST statistics (E-value, percent identity, query coverage) that provided high confidence in its identification, and did the BLASTx analysis reveal any conserved domains in the assembled proteins? Please add the regarding data to the text.*

We thank the reviewer for this suggestion. The key BLAST statistics and conserved domain information are already provided in the Results section of our manuscript. For the reviewer's convenience, we reiterate the data here:

- BLASTn analysis of the assembled contigs revealed high-confidence matches (E-value = 0.0, Query Coverage = 100%) with the following percent identities for each segment: RNA1: 96.13% to a Fujian isolate (OQ948458); RNA2: 96.38%; RNA3: 97.27%; and RNA4: 94.89% to Hangzhou isolates (NC_040402-NC_040404).

| Genomic Segment | Closest Isolate (Accession) | E-value | Percent Identity | Query Coverage |
| --- | --- | --- | --- | --- |
| RNA1 | Fujian (OQ948458) | 0.0 | 96.13% | 100% |
| RNA2 | Hangzhou (NC_040402) | 0.0 | 96.38% | 100% |
| RNA3 | Hangzhou (NC_040403) | 0.0 | 97.27% | 100% |
| RNA4 | Hangzhou (NC_040404) | 0.0 | 94.89% | 100% |

- Furthermore, BLASTx analysis confirmed the presence of conserved domains characteristic of TPNRBV, including methyltransferase-helicase (RNA1), helicase-RNA polymerase (RNA2), a virion membrane protein (RNA3-P24), and a movement protein (RNA4).

**line 119:**

*On the minimum contig length, what was the rationale behind choosing a 700 nucleotide (nt) minimum length for viral contigs? Wouldn't this filter potentially exclude smaller viral genes or genomes, and were any significant hits below this threshold observed and discarded?*

We thank the reviewer for raising this critical point regarding the potential exclusion of small viral agents.

The 700 nt threshold was employed as a conservative filter to prioritize high-confidence contigs for de novo assembly and analysis of conventional plant viruses. This heuristic is common in virome studies to reduce false positives from short, non-specific matches.

We fully agree with the reviewer that this filter would, in principle, exclude viroids and very small viral satellites. Therefore, to ensure a comprehensive survey, we performed an additional, dedicated analysis. Specifically, we conducted a separate BLASTn search of all assembled contigs and unassembled reads against the entire viral RefSeq database, including viroid sequences.

The results of this sensitive, unrestricted search confirmed that no significant hits (E-value < 1e-5) to viroids or other small viral agents were present in our samples. All significant viral signals corresponded to the two viruses we report: Tea plant necrotic ring blotch virus (TPNRBV) and Citrus-associated arlivirus 1 (CoAV1). Both of these viruses assembled into contigs well above our 700 nt cutoff (complete segments for TPNRBV and substantial contigs for CoAV1).

Thus, while the 700 nt filter served as a practical step for streamlining the focus on larger viruses, it did not lead to the loss of any biologically relevant small agents in this specific dataset. Our complementary unbiased screening validated the absence of viroids and confirmed that the viral community was dominated by TPNRBV and CoAV1, both successfully captured by our assembly pipeline. We have clarified this two-tiered analytical approach in the revised Methods section.

**lines 132-134:**

*Concerning the (de novo) assembly, please, write assembly algorithm and parameters (e.g., k-mer size, word size) which were used using the CLC Genomics Workbench, and parameters which were used for assessing the quality of the assembly (e.g., N50 contig length)*

Thank you for the comment. The *de novo* assembly was performed using the "De Novo Assembly" tool in CLC Genomics Workbench v20. The key parameters set for the assembly were: word size (for read overlap)= 20 and minimum contig length = 200 bp. The CLC assembler uses a proprietary algorithm, and as it is not a k-mer-based assembler, a k-mer size parameter was not applicable in this workflow.

The quality of the assembled viral contigs was assessed based on the following criteria: Contig Length & Coverage: All viral contigs were >700 bp and provided contiguous, full-length coverage of the four reference genome segments (RNA1-RNA4). High Sequencing Depth: The read coverage across all segments was deep and uniform (see Fig. 2a, b), with no regions below the minimum coverage threshold. Experimental Validation: The assembled sequences were validated by RT-PCR and Sanger sequencing of key genomic regions.

We note that the N50 statistic, commonly reported for de novo assembly of a complete transcriptome, was not a primary focus of our analysis. Our goal was specifically to assemble and validate the viral genomes from the complex plant transcriptome data. The most direct and relevant quality metrics for this viral-focused aim are the three criteria detailed above (contig completeness, depth, and experimental validation).

We will revise the Materials and Methods section to include the specific assembly parameters mentioned.

**lines** **132-134:**

*Regarding the assembly parameters, the length and similarity fractions for mapping were set to relatively low thresholds (0.5 and 0.8). What was the rationale for using these permissive parameters, and how did you ensure this did not lead to the misassembly of highly divergent or unrelated sequences?*

We thank the reviewer for raising this critical technical point. The reviewer is correct that the parameters (length fraction=0.5, similarity fraction=0.8) are relatively permissive.

The rationale for using these permissive parameters during the initial read-mapping phase was to maximize sensitivity for recovering viral reads from the complex host transcriptome, given the expected low abundance and potential genetic divergence of the local TPNRBV isolate. Using stricter thresholds at this stage could have prematurely excluded reads from genuinely divergent regions, biasing our assembly.

We ensured that this approach did not lead to misassembly through a stringent, multi-step validation pipeline:

1. Bioinformatic Filtering: All contigs were subjected to rigorous BLASTn analysis against viral databases (E-value < 1e-5) and filtered by a minimum length (≥700 nt).
2. Assembly Quality Control: The resulting genome assembly showed high-depth, uniform coverage across all segments (Fig. 2), with no regions falling below the minimum coverage threshold required for reliable SNV calling.
3. Experimental Validation: The assembled sequence was confirmed by independent RT-PCR and Sanger sequencing of multiple genomic regions, as detailed in the 'Verification of HTS data' section.
4. Biological Validation: The logical and well-supported placement of our isolates within the TPNRBV phylogeny further confirms the biological relevance of our assembly.

Therefore, the permissive mapping served as a sensitive initial 'net,' while all subsequent steps acted as specific 'filters.' This is a recognized strategy in viral metagenomics for identifying novel or divergent variants.

In direct response to the reviewer's comment, we have revised the 'De novo assembly' subsection in the Methods to explicitly state the rationale for these parameters. The updated text now reads:

"Given the low abundance of viral RNA within the host transcriptome and to accommodate potential genetic divergence of the local isolate, permissive alignment parameters were used to maximize viral read recovery."

We believe this addition, along with the existing multi-step validation described in our manuscript, provides a complete and clear justification for our bioinformatic strategy.

**line 136:**
*The minimum variant frequency was set to 1% (0.01), which is very sensitive. Given that the RNA was pooled from 20 different plants, how can you distinguish between a true low-frequency variant within one plant's viral population and a variant that is fixed but only present in a small subset of the infected plants in the pool? Please consider such limitations in conclusion.*

We thank the reviewer for highlighting this important limitation of the pooled-sample design. To address this point transparently, we have added the following clarification to the Materials and Methods section in the paragraph describing SNV prediction: *"It is important to note that, as the RNA-seq data originated from a pool of 20 plants, the identified SNVs and their frequencies represent a composite of viral sequences from the entire sampled population. Therefore, a low-frequency variant could represent either a true minor variant within a single host's viral population or a fixed variant in a virus infecting only a small subset of the plants in the pool."* This limitation is also now acknowledged in the revised Discussion.

**line 137:**

*Beyond the statistical p-value, were any of the identified non-synonymous SNVs, especially in key genes like P22, validated by an independent method like Sanger sequencing to rule out potential sequencing or mapping artifacts? Add them to the text.*

We thank the reviewer for raising this important point regarding validation. We have added an explicit acknowledgment of this limitation to the new "Considerations on the Pooled-Sample Design" paragraph in the Discussion section: **"**Furthermore, the SNVs reported here were identified bioinformatically; their independent validation by methods such as Sanger sequencing remains a necessary step for future work." This clarifies that the SNV calls are preliminary and require future experimental confirmation.

**lines 175-176:**

*On the sequencing output, please, add the total sequencing output (e.g., total gigabytes of data or number of raw reads generated) for each of the pooled libraries.*

High-throughput sequencing of the two libraries yielded a total of 38,125,708 and 103,830,264 raw paired-end reads for the 2021 and 2022 samples, respectively.

**lines 175-177:**

*The 2022 library generated nearly 15 times more total contigs than the 2021 library (172,521 vs 11,638), despite having only about 2.5 times more clean reads. What factors account for this massive difference in assembly complexity? Could it be due to a higher diversity of other microorganisms (e.g., fungi, bacteria) in the 2022 samples, or different assembly parameters? Please discuss these results in the text.*

We sincerely thank the reviewer for this critical observation, which allowed us to correct significant reporting errors and improve the clarity of our results. The reviewer is absolutely correct to question the discrepancy. Upon thorough re-examination of our assembly output, we discovered and corrected two reporting inaccuracies in the original manuscript: (1) The total number of contigs from the de novo assembly of the 2022 library is 34,454, not 172,521, and (2) The number of TPNRBV-specific contigs identified in the 2022 library is 36, not 30. We apologize for these errors. The correct values have been updated in the revised manuscript (*De novo* transcriptome assembly of the unmapped reads resulted in 11,638 contigs for library 2021 and 34,454 contigs for library 2022).

With the corrected values, the data present a coherent and technically expected picture. The total number of contigs increased approximately threefold from 2021 to 2022 (34,454 vs. 11,638). This is directly proportional to the ~2.7-fold increase in clean reads available for assembly (25.6 million vs. 10.86 million), as now correctly reported. Therefore, the primary factor accounting for the difference in assembly output is the larger volume of high-quality sequencing data in the 2022 run, which allows the assembly algorithm to resolve more transcript fragments into distinct contigs. This reflects normal technical variation between independent library preparations and sequencing depths.

We have added the following sentences to the Results section to incorporate this explanation: "The approximately threefold increase in the total number of contigs assembled from the 2022 library compared to the 2021 library (34,454 vs. 11,638) is consistent with the proportional increase in clean sequencing data available for assembly (~2.7-fold higher), reflecting expected technical variations between sequencing runs. The number of TPNRBV-specific contigs was similar in both libraries (36 vs. 35), confirming the consistent and dominant presence of the virus in the sampled pools."

We believe these corrections and the added discussion resolve the apparent anomaly and provide an accurate, transparent report of the assembly data. We are grateful for the reviewer's meticulous attention to detail.

**Reviewer #2, Comment on lines 179-180:**

*The text states the (majority) of contigs were associated with TPNRBV. This implies a significant number of non-TPNRBV contigs were also assembled. What were the other top BLASTn hits for these non-TPNRBV contigs? Were any other novel or known plant viruses identified in these libraries? Please add these data and their discussion.*

Thank you for raising this important point. As mentioned, the majority of the assembled contigs were associated with TPNRBV. Regarding the other contigs, a detailed analysis was performed to identify any potential novel or known plant viruses.

By applying stringent filtering criteria (contig length ≥ 700 nucleotides, strong homology in BLASTn, and meaningful coverage of viral genomic regions), none of the other contigs provided reliable evidence for the presence of an additional plant virus in these libraries.

The non-TPNRBV contigs can be categorized as follows:

1. **Contigs showing partial homology to other plant viruses:** A number of medium-length contigs (200-700 nucleotides) showed matches to fragments of other plant viral sequences in BLASTn. However, these matches were very short, covered only a minor part of the viral genome, and did not meet our stringent criteria for confident virus identification.
2. **Contigs of non-plant viral origin:** Several contigs showed homology to sequences from phages or mycoviruses. These are most likely associated with the plant's microbiome and are not considered plant pathogens.

**Very short contigs (less than 200 nucleotides):** As is common in HTS-based virome studies, a number of short contigs were assembled. Although a few showed sporadic viral matches, their length is insufficient for reliable virus identification, carries a high risk of false positives, and therefore they were not considered significant.

Consequently, after implementing our rigorous bioinformatic pipeline, TPNRBV (and CoAV1, as mentioned in the Discussion section) were confirmed as the only plant viruses robustly detected in these tea transcriptome libraries. To ensure transparency, we have revised the Results section to clarify this point:

BLASTn analysis showed that the majority of the contigs were associated with TPNRBV in both libraries. The remaining non-TPNRBV contigs were examined using stringent criteria (contig length ≥ 700 nucleotides and meaningful coverage of viral genomic regions in BLASTn). While some showed short, partial homology to other plant viruses or to non-plant viral sequences (e.g., phages or mycoviruses likely associated with the plant microbiome), none met the thresholds for confident identification of an additional plant virus. Therefore, TPNRBV was confirmed as the dominant and robustly detected plant virus in these transcriptome libraries.

Therefore, after applying stringent filters based on match quality, meaningful length, and relevance to the host plant, no other contigs indicating the presence of a novel or known plant virus with high confidence were identified in our libraries. For transparency, this point has been revised in the Results section of the manuscript.

**lines 180-182:**

*Although the total number of contigs differed greatly between libraries, the number of TPNRBV-related contigs was similar (35 vs 30). What was the average length and depth of coverage of these viral contigs in each library? Did the 2022 library, with more data, produce more complete or higher-quality TPNRBV contigs? Please discuss them in the text.*

We thank the reviewer for this insightful question. The reviewer is correct that the number of TPNRBV-specific contigs was similar between libraries (35 in 2021 vs. 36 in 2022), despite the larger amount of total data in 2022. This indicates that the virus was the dominant and consistently assembled agent in both pools.

Regarding contig quality:

- Average length: The viral contigs in both libraries assembled into complete genomic segments. The final, polished genome sequences were identical in length (RNA1: 5922 nt, RNA2: 4107 nt, RNA3: 2678 nt, RNA4: 2272 nt), indicating that both datasets yielded complete viral genomes.
- Depth of coverage: The key difference was in the depth of sequencing support. The mapped read count for each segment was 4 to 5 times higher in the 2022 library (e.g., 3.8 million vs. 0.8 million reads for RNA1). This substantially higher depth did not produce longer contigs (as the genomes were already complete) but increased the confidence and robustness of the assembly, particularly for resolving low-frequency variants (SNVs) and ensuring no regions were under-sampled. The higher coverage in 2022 is also reflected in the greater number of total signatures identified per segment (41 in 2022 vs. 20 in 2021).

In summary, the 2022 library, with its greater sequencing depth, did not generate more or longer viral contigs but provided a much higher-resolution dataset. This increased depth strengthened the reliability of our consensus genome sequences and significantly enhanced the sensitivity and statistical power of our downstream population genetic analysis (SNV detection).

**lines** **180-186:**

*The TPNRBV genome has four segments. Were the coverage and depth of reads uniform across all four reference segments (RNA1-RNA4) during the mapping process, or were there regions with significantly lower coverage that might have been missed in the assembly and SNV analysis? Please add relates results to the text.*

We thank the reviewer for raising this critical point about coverage uniformity. We have now added a dedicated paragraph to the Results section to address it. The added text is:

(The depth of coverage across the four genomic segments was not uniform, a common observation in transcriptomic studies of multipartite RNA viruses. Despite this variation, the coverage maps (Fig. 2a, b) confirm that the entire length of all segments was covered, with no regions falling below the minimum coverage threshold of 2X required for reliable SNV detection. This ensures the completeness of the genome assemblies and the robustness of the variant analysis)

As the reviewer correctly anticipated, read coverage was not perfectly uniform across the four RNA segments, which is a common feature in RNA-seq studies of multipartite viruses.

Despite this variation, our coverage maps (Fig. 2a, b) confirm that the entire length of all four genomic segments was covered in both libraries. Crucially, no region exhibited coverage below the minimum threshold of 2X that we established for reliable SNV calling in our bioinformatic pipeline (see Methods). Therefore, while the depth fluctuated, the assembly of complete genomes and the subsequent SNV analysis are robust and not biased by missing or under-represented genomic regions."

**lines 191-195:**

*Considering the experimental design: Given that the samples were pooled, how can the study confidently attribute specific genetic variations (like the SNVs mentioned in the abstract) to individual viral isolates, rather than treating them as a mixture from a viral population?*

We sincerely thank the reviewer for this critical point. We have thoroughly revised the manuscript to consistently frame our findings at the population level. Specifically:

1. In the Results section, we have changed the title of the SNV discovery to "Identification of sequence variants within the TPNRBV population" and rewritten the opening paragraph to explicitly state that variants were called from pooled data, removing any reference to within-plant "quasispecies".
2. Similarly, the gene expression section is now titled "Population-level expression profile of TPNRBV ORFs" and its opening clarifies that expression levels were assessed "across the infected plant population" using pooled data.
3. All subsequent interpretations in the Discussion have been reframed to reflect population-level, not within-host, signals.

**lines 193-196:**

*The identified non-synonymous SNVs have a very broad frequency range (0.26% to 21.47%). How are these SNVs distributed across this range? Are most SNVs at very low frequency (e.g., below 2%), suggesting they are transient mutations, or is there a significant number at higher frequencies, suggesting they may be selectively advantageous? It would be more informative adding these data to the results and discussion sections.*

Thank you for the suggestion. We have added the requested distribution analysis to the Results section: "The distribution of these frequencies was highly skewed: 90 out of 93 SNVs (96.8%) occurred at frequencies below 5%, consistent with a background of transient mutations. Only three variants were found at frequencies above 5% (5.76%, 14.44%, and 21.47%)."

We have also added a corresponding interpretation in the Discussion. We state that the three high-frequency outliers "may represent variants under different selective pressures or founder effects within the population, warranting further investigation." This directly addresses the reviewer's question about potential selective advantage, while acknowledging that our population-level data cannot definitively distinguish between selection and other demographic effects.

**lines 198-201:**

*For the non-synonymous SNVs found at the highest frequencies (e.g., >10%), did in silico protein analysis (like SIFT or PROVEAN) predict whether these amino acid changes would be deleterious, neutral, or beneficial to the protein's function? It would be more informative adding these data to the results and discussion sections*

We thank the reviewer for this suggestion. We did not perform *in silico* pathogenicity predictions (e.g., with SIFT or PROVEAN) as part of this initial genomic discovery study. We fully agree that such analysis on the high-frequency SNVs is a logical and important next step. To explicitly state this future direction, we have now added a dedicated "Limitations and Future Perspectives" section to the manuscript. In this new section, we state:
"A critical next step is the functional prioritization of the identified SNVs. Specifically, the three high-frequency non-synonymous variants (at 5.76%, 14.44%, and 21.47%) represent prime candidates for in silico predictive analysis using tools such as SIFT or PROVEAN to assess whether their amino acid changes are deleterious, neutral, or potentially advantageous."

**line 199-201:**

*RNA1 and RNA2 harbor the vast majority (81 out of 93) of the non-synonymous SNVs. Does the high mutation load correlate with known variable domains (e.g., the RNA-dependent RNA polymerase in RNA1), suggesting potential escape mutants or adaptability? Please enter it to the discussion.*

"We thank the reviewer for raising this important point about the functional interpretation of the mutation distribution. As noted in our initial response, we have added a sentence to the Results section to explicitly state the uneven distribution of variants: *'This uneven distribution resulted in a high density of non-synonymous variants in the replicase genes encoded by RNA1 and RNA2, while the movement protein (RNA4) and most ORFs of RNA3, including the highly expressed P22 gene, showed remarkably low variant counts.'

Furthermore, we have now significantly expanded the Discussion to directly address the reviewer's specific question about variable domains and adaptability. We note that the concentration of SNVs in RNA1/RNA2 aligns with the replicase complex being a known hotspot for variation in RNA viruses (e.g., the error-prone RdRp). However, we contextualize this finding by emphasizing that our pooled-sample design reveals population-level diversity—likely representing multiple circulating lineages—rather than providing definitive evidence for within-host adaptive evolution or escape mutants. A new passage in the Discussion now reads: 'The high density of non-synonymous variants in the replicase genes likely reflects both the inherent genetic plasticity of these regions and the presence of multiple similar TPNRBV lineages in the surveyed population, rather than conclusive evidence for within-host adaptive evolution.

We believe these revisions fully address the reviewer's insightful comment.

**lines 200-201:**

*The 15 SNVs shared between the 2021 and 2022 collections are highly interesting. What are the specific amino acid changes and which proteins are affected by these shared, conserved mutations? Does their conservation across seasons suggest they confer a selective advantage in the Iranian tea plant environment? Results and discussion on this topic in the manuscript would be informative and interesting.*

We thank the reviewer for these insightful questions regarding the 15 shared SNVs.

1. Specific Amino Acid Changes and Affected Proteins: As detailed in Supplementary Table 1, all 15 conserved non-synonymous SNVs are located in the replicase genes—specifically, within the Methyltransferase-Helicase (RNA1) and Helicase-RNA polymerase (RNA2) open reading frames. The table provides the exact nucleotide change (e.g., A→T) and resulting amino acid substitution for each variant. As noted in the manuscript, these proteins are central to viral replication.
2. Interpretation and Potential Selective Advantage: We have added the following sentence to the Discussion to address the potential significance of this conservation: "**The 15 SNVs conserved between the 2021 and 2022 pools are of particular interest, as their persistence may indicate a potential selective advantage or founder effect in the local TPNRBV population; their functional impact merits further investigation**."

Their conservation across two growing seasons is notable. The concentration of these shared mutations in the replicase complex suggests they could influence core viral functions. While our data cannot definitively distinguish between positive selection for a local adaptation and a founder effect, their persistence makes them prime candidates for functional studies. We have now explicitly framed this as a key objective for future research.

**lines 140-141& & 203-204:**

*The SNVs were visualized on 3D protein structures. For the non-synonymous SNVs identified, did their locations on the 3D structure suggest a potential functional impact (e.g., were they located in active sites, binding interfaces, or stable domains)? What was the key structural insight gained? Please discuss these results in the manuscript.*

*The analysis showed "no visible changes" in the 3D structure. What was the resolution of this analysis? Was it a simple visual inspection, or were quantitative metrics (e.g., changes in binding pocket volume, electrostatic surface potential, or protein stability) calculated? Could the mutations affect properties not visible in a static structure, like protein flexibility or interaction networks? Adding these data will contribute to the scientific content of the manuscript.*

We thank the reviewer for this insightful question. We have expanded the description in the Results section to clarify the scope and limitations of our structural analysis. Following the statement about no visible changes, we now add: "This structural comparison provided a preliminary, qualitative assessment based on visual inspection of the superimposed models. Quantitative analyses of biophysical properties—such as changes in binding pocket volume, electrostatic surface potential, or protein stability—were not performed, as they require specialized molecular dynamics simulations or energy calculations that were beyond the primary genomic focus of this study. It is therefore possible that the identified mutations could affect functional properties not discernible in a static structural alignment". This explains that our analysis was a first-order visualization and that deeper functional insights into the potential impact of the SNVs would require more advanced computational studies.

**lines 206-209:**

*Regarding expression normalization: The study uses TPM for normalization. Was the "total mapped reads" used in the TPM denominator the total number of reads that mapped specifically to the TPNRBV genome, or was it the total number of reads in the entire sequencing library (including plant and other microbial reads)?*

Response: We thank the reviewer for raising this important point. In our analysis, TPM values were calculated exclusively based on reads mapped to the TPNRBV reference genome, not on the total number of reads in the entire sequencing library. Specifically, trimmed reads were mapped to the TPNRBV RefSeq, and TPM normalization was performed using the total number of virus-mapped reads as the denominator. This approach allows for a valid comparison of relative expression levels among TPNRBV ORFs within the viral transcriptome, independent of host or other microbial RNA abundance. Also, we clear this in the material and method section.

**lines 229-233:**

*The phylogenetic trees for the different RNA segments were discordant (RNA1 vs. RNA2-4). The discussion states a close relationship to Hangzhou isolates but doesn't address this mixed signal. How do you interpret this conflicting phylogenetic history in the absence of more detectable recombination? Also, the discordant phylogenetic trees for RNA1 (groups with Fujian) versus RNA2-RNA4 (groups with Hangzhou) is a critical finding that is buried in the results and not adequately discussed. The discussion should explicitly address this and hypothesize about its cause, especially since recombination was weak. It would be better to mention these issues in the discussion*.

We agree with the reviewer that this discordant phylogenetic signal is a critical finding that warrants prominent discussion. To ensure it receives the emphasis it deserves, we have elevated its interpretation from a descriptive report in the Results to a dedicated and explicit discussion in the Discussion section (However, an intriguing finding of our phylogenetic analysis was the incongruent placement of the Iranian isolates: they clustered with isolates from Fujian province in the RNA1 tree, but with the Hangzhou isolate in the trees for RNA2, RNA3, and RNA4 (Fig. 5). Since no evidence of intra-segment recombination was detected using RDP4, this pattern is most parsimoniously explained by genomic reassortment, a common evolutionary mechanism for viruses with multipartite genomes [19]. This suggests that the Iranian TPNRBV isolates could be natural reassortants, possibly originating from a mixed infection of two parental lineages circulating in China—one lineage related to the Fujian isolates (donating RNA1) and another related to the Hangzhou isolate (donating RNAs 2-4). The subsequent long-distance movement of this reassortant strain to Iran could have occurred through the exchange of infected plant material. While convergent evolution or insufficient sequence diversity in public databases could also contribute to such topological discrepancies, reassortment remains the most direct explanation for the observed segment-specific clustering. This hypothesis highlights the potential for reassortment to generate genetic novelty in TPNRBV and underscores the importance of obtaining full-genome sequences from diverse geographical regions to understand the segment exchange dynamics and global spread of this emerging virus). In this new paragraph, we highlight the segment-specific pattern and propose genomic reassortment between Chinese parental lineages (Fujian-like for RNA1 and Hangzhou-like for RNAs 2-4) as the most parsimonious evolutionary hypothesis, given the absence of detectable intra-segment recombination.

**Lines 245-248:**

*The Iranian isolates share a pattern of conserved 5' terminal motifs with the Hangzhou isolate (absent in RNA2-RNA4, a specific variant in RNA1). Do you have data about the critical role of these motifs for viral replication? Does their shared, atypical structure provide stronger evidence for a direct evolutionary link than the nucleotide identity percentages? Enter this data for clarifying importance of the conserved terminal motifs.*

Thank you for highlighting this important feature. We confirm that the conserved 5′-terminal motif is absent in RNA2-RNA4 but present in RNA1 of both the Iranian and Hangzhou isolates. As noted in prior studies on TPNRBV (Maruyama et al. [9]), such conserved terminal motifs are considered significant for the replication and packaging of segmented RNA viruses. The shared, specific loss of this motif in three segments (RNA2-RNA4) of both isolates while it is retained in RNA1 constitutes a synapomorphy (a derived trait inherited from a common ancestor). This pattern provides a more robust and specific evidence for a direct evolutionary link between these two isolates than general nucleotide identity percentages alone, as such a precise, multi-segment loss is unlikely to occur independently.

*The following text has been added to the manuscript in response to this comment*

Similar to isolates from Hangzhou, TPB-Iran and TPNRBV-Ir lacked these conserved sequences in RNA2, RNA3, and RNA4. However, such as TPNRBV_HZ, RNA1 of TPB-Iran and TPNRBV-Ir contained the sequence 5'-ATTAACGA-3', along with five additional nucleotides before the conserved motif (5'-TGGGGATTACGA-3').

This shared, specific pattern of motif loss in RNA2-RNA4 coupled with its conserved (and extended) presence in RNA1 constitutes a notable synapomorphy (a derived trait indicative of common ancestry) between the Iranian and Hangzhou isolates. While prior studies suggest such terminal motifs are significant for viral replication and packaging in segmented RNA viruses [9, 16], this precise, multi-segment deletion is unlikely to have arisen independently. Therefore, this structural signature provides stronger evidence for a direct evolutionary link between these isolates than nucleotide identity percentages alone.

**Lines 250-253:**

*The 110-nt insertion in RNA4 is a unique marker shared only by the Iranian and Hangzhou isolates. Does this insertion occur in a coding or non-coding region? What is its predicted functional impact, and does its presence override the phylogenetic signal from RNA1, making a Hangzhou origin more likely for the majority of the genome? This discussion would add the significance of the RNA4 insertion.*

We thank the reviewer for this insightful question regarding the unique 110-nt insertion.

To address the reviewer's specific points:

1. Location: The insertion is located in the 3′ non-coding region (UTR) of RNA4.
2. Functional Impact: As it resides in a non-coding region, its primary impact is likely on RNA secondary structure, stability, or replication efficiency, rather than direct protein function. We will add a sentence to the Discussion noting that such insertions can influence viral fitness through these mechanisms, presenting a hypothesis for experimental validation.
3. Phylogenetic Conflict: No, its presence does not override the phylogenetic signal from RNA1. The RNA1 tree robustly groups the Iranian isolate with Fujian isolates. The shared insertion with Hangzhou in RNA4 is best interpreted as a shared derived trait (synapomorphy) that likely arose once in a common ancestor of these two isolates after they diverged from the Fujian lineage, or through a rare horizontal transfer event. It does not contradict the reassortment hypothesis (Fujian-like RNA1 + Hangzhou-like RNAs 2-4).

Action Taken: We have revised the Discussion to incorporate these points. The added text clarifies the insertion's non-coding location, discusses its potential evolutionary significance as a molecular marker, and explicitly states that it does not conflict with the RNA1 phylogenetic signal, thereby strengthening the narrative of a complex evolutionary history involving both reassortment and unique indel events.

**Lines 257-260:**

*The study concludes the mapped reads were sufficient for full genome coverage. What was the minimum depth of coverage across the entire TPNRBV genome in each library, and were there any regions with consistently low coverage that might be prone to assembly errors?*

Response: We thank the reviewer for this important question. In both RNA-seq libraries (2021 and 2022), the entire TPNRBV genome was covered with a minimum depth of ≥2×, which was also the threshold used for reliable SNV detection. Although read coverage was not uniform across the four genomic segments—an expected feature of multipartite RNA viruses—no genomic region consistently fell below this minimum coverage level. Coverage plots (Fig. 2a,b) confirm continuous coverage across all segments, with no gaps or systematically low-coverage regions that would be prone to assembly artifacts. The substantially higher sequencing depth in the 2022 library further increased confidence in genome completeness and assembly accuracy. Also we clear this in the result section about fig 2.

**Lines 267-271:**

*The SNV data indicates a diverse quasispecies, while the phylogenetic trees show low variability. Given that the pooling strategy is the most likely cause, what specific follow-up experiment did you use to definitively resolve whether this diversity represents a true quasispecies within single plants or a mixture of very similar but distinct isolates across the plantation? Please clear this Quasispecies issue.*

We agree with the reviewer. The apparent diversity is indeed due to our pooling strategy, not proof of a within-host quasispecies. To definitively resolve this would require a different experiment (e.g., deep sequencing of individual plants), which was beyond the scope of this field-level genomic survey. Accordingly, we have removed all references to "quasispecies" and reframed our conclusions throughout the manuscript to reflect strictly population-level diversity (i.e., a mixture of similar isolates circulating in the plantation). A new paragraph in the Discussion explicitly details this Considerations of the pooled-sample design.

Considerations on the Pooled-Sample Design

The interpretations of this study are framed by its pooled-sample design. While this approach efficiently captures population-level diversity and expression profiles, it has inherent limitations. First, the SNV frequencies reported represent a composite from multiple plants; a variant at 1% frequency could be a rare mutation in one host's viral population or a fixed difference in a virus infecting 1 out of 20 plants. Consequently, our data describe the genetic structure of the TPNRBV population at the field level but cannot delineate within-host evolutionary dynamics. Second, the TPM values reflect average viral gene expression across all infected tissue in the pool and should not be interpreted as temporal expression changes within a single infection. Furthermore, the SNVs reported here were identified bioinformatically; their independent validation by methods such as Sanger sequencing remains a necessary step for future work. Despite these constraints, our study provides valuable insights into the population genomics and expressed repertoire of TPNRBV in Iranian tea plantations, establishing a foundation for more detailed within-host studies in the future.

**Lines 272-273:**

*Were the 93 non-synonymous SNVs evenly distributed across the four segments of the TPNRBV genome (RNA1-RNA4), or were they concentrated in specific segments or genes (e.g., the highly expressed P22 gene)? What might a cluster of mutations in one particular gene suggest? Please add data regarding these questions to the results and discussion sections.*

We thank the reviewer for raising this important point about SNV distribution. Please find our point-by-point response below.

**1. Distribution Across Genome Segments:**
The 93 non-synonymous SNVs were not evenly distributed across the four genomic segments. They were highly concentrated in the replicase segments: 44 SNVs were identified in RNA1 and 37 in RNA2. In stark contrast, only 1 SNV was found in RNA3 and 1 in RNA4. This data has been clearly stated in the Results section of our revised manuscript.

**2. Status of the P22 Gene:**
Consistent with the overall low variation in RNA3, no SNVs were detected in the highly expressed P22 gene at our 1% frequency threshold in the pooled sample analysis.

**3. Interpretation and Added Discussion:**
We have added a dedicated analysis to the Discussion section to interpret this pattern. We propose that the high density of mutations in RNA1 and RNA2 (encoding the viral replicase) reflects the inherent genetic plasticity of these regions, which are often associated with error-prone replication and may tolerate more variation. This clustering could provide a reservoir of genetic diversity for potential adaptation. Conversely, the near absence of SNVs in genes like P22 and the movement protein (RNA4) suggests they may be under stronger functional or structural constraints, making them more conserved. This interpretation strengthens the evolutionary narrative of our study by linking genetic variation to potential gene function.

We believe these additions fully address the reviewer's questions regarding SNV distribution and its implications.

**Lines 290-291:**

*For the highly expressed P22 gene, what was the average read depth of coverage across its ORF? Was the read coverage uniform, or were there regions with unusually high or low coverage that might suggest post-transcriptional processing or assembly artifacts? Please add and discuss related results*.

Response: We appreciate the reviewer’s focus on the P22 gene. Read mapping results showed that the P22 ORF was covered continuously across its entire length in both RNA-seq libraries. While coverage depth varied along the genome, as expected for viral transcriptomic data, **no regions within the P22 ORF exhibited consistently low coverage or abrupt coverage drop-offs**, and coverage did not fall below the minimum threshold (2×) used for variant detection.

The coverage profile of P22 also did not show localized spikes or discontinuities that would be indicative of assembly artifacts or post-transcriptional RNA processing. In addition, no SNVs were detected in the P22 ORF at the 1% frequency threshold, further supporting the accuracy and stability of its assembly. Together, these observations indicate that the high TPM values observed for P22 reflect genuine high transcript abundance rather than technical or assembly-related artifacts.

**Lines 292-298:**

*The information on gene expression is unnecessarily duplicated here. It would also be appropriate to mention the reason why the P22 and P14 genes are attributed the function of virus-host interaction and RNA silencing suppression, respectively.*

We thank the reviewer for these valuable suggestions.

1. **Removal of redundancy:** We have removed the redundant sentences regarding the high expression and unclear function of P22/P14.
2. **Justification for functional speculation:** As suggested, we have provided a clear rationale. The hypothesis regarding P22 is now based on (a) its high expression level in our data, and (b) the established role of highly expressed small proteins as RNA silencing suppressors (VSRs) or host interaction factors in other plant virus systems [Burgyán and Havelda, 2011].
3. The revised paragraph now reads: " The expression levels of TPNRBV ORFs were also examined using RNA-seq data, with the highest expression observed for P22, followed by P24 and P14. Although the functions of P22 and P14 remain unclear, the notably high expression of P22 suggests it may play a significant role in the infection cycle. In many plant viruses, highly expressed accessory proteins often function as RNA silencing suppressors (VSRs) or facilitate host-virus interactions [18]. Based on its high expression and this established functional analogy [Burgyán and Havelda, 2011], we hypothesize that P22 is a prime candidate for such functions in TPNRBV. This presents a testable hypothesis for future studies."We believe these edits have eliminated the duplication and provided the necessary logical foundation for the functional discussion.

Burgyán J, Havelda Z. Viral suppressors of RNA silencing. Trends Plant Sci. 2011;16(5):265-72. doi: 10.1016/j.tplants.2011.02.010. Epub 2011 Mar 24. PMID: 21439890.

**lines 304-305:**

*The statement that P22 has "no SNVs" should be nuanced. It would be more accurate to state that “no SNVs were found (above the 1% frequency threshold in this particular sample pool)”. This leaves open the possibility of lower-frequency variants or variation in other populations.*

We thank the reviewer for this precise correction. We have amended the statement in the Results section to: **"...**and notably, no SNVs were identified in the P22 gene located in RNA3 at the 1% frequency threshold in this pooled dataset." Furthermore, in the Discussion, we have refined the related conclusion to: "The conservation of the P22 gene (with no variants above 1% in our pooled data) makes it a prime target for developing robust diagnostic tools for TPNRBV."

**lines 306-308:**

*Given the high similarity to Chinese isolates, the mixed phylogeny, and the shared unique insertion, what is the most plausible hypothesis for the emergence of TPNRBV in Iran? Does the evidence point more strongly to a single introduction event from a population with mixed ancestry, or multiple independent introductions of different segments? It is recommended that this issue be discussed by including more data in addition to presenting the phylogeny trees.*

We thank the reviewer for raising this crucial question regarding the origin and emergence of TPNRBV in Iran. We agree that our phylogenetic and genomic data provide a strong basis for formulating a testable hypothesis.

In response, we have added a dedicated paragraph to the Discussion section (Considering the high sequence similarity, the congruent reassortment pattern in both Iranian isolates, and their shared unique 110-nt insertion—a marker unlikely to arise independently—the most parsimonious hypothesis for the emergence of TPNRBV in Iran is a single introduction event. This likely involved infected plant material carrying a virus population with mixed ancestry (or a pre-existing reassortant) from China, followed by its local establishment and spread. The evidence does not strongly support a scenario of multiple, independent introductions of different genomic segments) that directly addresses the reviewer's question. Based on the congruent evidence – specifically, the high sequence similarity to Chinese isolates, the identical reassortment pattern (RNA1 from Fujian-related and RNAs 2-4 from Hangzhou-related lineages) observed in both Iranian isolates, and their shared unique 110-nt insertion in RNA4 – we conclude that the most parsimonious explanation is a single introduction event. This event likely involved the movement of infected plant material carrying a virus population with mixed ancestry (or a pre-existing reassortant) from China into Iran, followed by local establishment. The consistency of these multiple, independent genomic signatures in both of our Iranian isolates makes a scenario of multiple, independent introductions of different genomic segments less plausible.

We believe this addition significantly strengthens the discussion of our findings and provides a clear evolutionary hypothesis for the virus's arrival in Iran, as suggested by the reviewer.

**lines 306-312:**

*The Iranian isolates group with Fujian in the RNA1 tree but with Hangzhou in the RNA2-RNA4 trees. If RDP4 detected no recombination, what other evolutionary mechanisms could explain this apparent reassortment of genomic segments? Does this suggest the Iranian isolates could be a natural reassortant between two different Chinese TPNRBV lineages? Please enter these points in the discussion which will contribute to the article’s appeal and scientific clarity.*

We thank the reviewer for this insightful question, which raises a key point about the evolution of this multipartite virus.

As suggested, we have added a dedicated paragraph to the Discussion section to address these points. The added text is as follows:

"An intriguing finding of our phylogenetic analysis was the incongruent placement of the Iranian isolates: they clustered with isolates from Fujian province in the RNA1 tree, but with the Hangzhou isolate in the trees for RNA2, RNA3, and RNA4 (Fig. 5). Since no evidence of intra-segment recombination was detected using RDP4, this pattern is most parsimoniously explained by genomic reassortment, a common evolutionary mechanism for viruses with multipartite genomes [19]. This suggests that the Iranian TPNRBV isolates could be natural reassortants, possibly originating from a mixed infection of two parental lineages circulating in China—one lineage related to the Fujian isolates (donating RNA1) and another related to the Hangzhou isolate (donating RNAs 2-4)."

To directly answer the reviewer's question: yes, in the absence of recombination, the most plausible evolutionary mechanism to explain the phylogenetic incongruence is indeed genomic reassortment.  Therefore, our data are consistent with the hypothesis that the Iranian isolates are natural reassortants between two different Chinese lineages (Fujian-like and Hangzhou-like). While other explanations like convergent evolution cannot be entirely ruled out, reassortment is considered the most parsimonious explanation given the multipartite nature of the viral genome and the absence of intra-segment recombination signals. This addition enhances the article's scientific clarity by explicitly discussing this plausible evolutionary scenario.

**Reviewer #3**

• *Vector/transmission statements. The text mentions mechanical and seed transmission and hypothesized insect vectors (mites, aphids, leafhoppers, whiteflies). Please align these statements with current evidence for kitavirids/blunerviruses (e.g., Brevipalpus mites for related viruses) and cite primary sources. If evidence is lacking for TPNRBV, say so explicitly.*

We thank the reviewer for this important request for clarity on transmission vectors. We have revised the Background section accordingly.

1. For related viruses in the family *Kitaviridae*: The text now explicitly cites primary sources (e.g., Robinson et al., 2016; Tassi et al., 2025) confirming that eriophyid and tenuipalpid mites (e.g., *Brevipalpus* spp.) are established vectors for other kitaviruses.

2. For TPNRBV specifically: The revised text now states clearly: "To date, no specific insect or mite vector has been identified for TPNRBV." The hypothesized role of other insects (aphids, etc.) is now presented strictly as a suggestion for future research, given transmission modes in other plant virus families.

3. Our experimental evidence: As noted, reference [4] (our previous work) provides the direct experimental evidence for mechanical and seed transmission cited in the manuscript.

These edits ensure our transmission statements are precise, evidence-based, and distinguish clearly between established facts for related viruses and the current unknowns for TPNRBV.

Robinson TS, Scherm H, Brannen PM, Allen R, Deom CM. Blueberry necrotic ring blotch virus in southern highbush blueberry: Insights into in planta and in-field movement. Plant Dis. 2016; 100, 1575–1579.

Tassi AD, Ramos-González PL, Flechtmann CHW, Amrine JW Jr, Sarkhosh A, Freitas-Astua J, Kitajima EW, Marques JPR, Harmon PF, Carrillo D. Eriophyid Mites Vector the Kitavirus Blueberry Necrotic Ring Blotch Virus: Insights into the Viral Transmission and Its Infection on Blueberry Plants. Phytopathology. 2025 Aug;115(8):1038-1050. doi: 10.1094/PHYTO-02-25-0063-R. Epub 2025 Aug 2. PMID: 40323349.

• *Pooled samples and interpretation. Because samples were pooled by year, allele frequencies reflect mixtures of infections across plants, not clonal within-host dynamics. Likewise, TPM differences from pooled RNA-seq cannot be interpreted as within-host expression changes. Please reframe conclusions accordingly (no linkage inference; population-level, not within-host, signals).*

We sincerely thank the reviewer for this fundamental comment. We have thoroughly reframed the entire manuscript to consistently reflect **population-level**, not within-host, signals. Key changes include: (1) Renaming and rewriting the SNV and gene expression sections in Results to emphasize "population-level" data; (2) Removing all references to "quasispecies" and within-host dynamics; (3) Adding a dedicated paragraph in the Discussion titled **"Considerations on the Pooled-Sample Design"** that explicitly explains the limitations regarding SNV frequency interpretation and TPM values, stating they represent composite, population-level averages and cannot be used to infer within-host dynamics.

• *PCR verification details. Provide primer sequences (TPNRBV3-F/R, MP-F/R), exact annealing temperatures (rather than “respective temperatures”), and the counts of PCR-positive plants per pool. This will improve reproducibility.*

We thank the reviewer for the suggestion to enhance methodological clarity and reproducibility. We have updated the manuscript accordingly.

1. **Primer Sequences and Annealing Temperatures:** The exact nucleotide sequences of the primers TPNRBV3-F/R and TPNRBV-MP-F/R are now explicitly provided in the 'Verification of HTS data' subsection (Materials and Methods). The precise annealing temperatures used for each primer pair (54 °C for TPNRBV3-F/R and 50 °C for TPNRBV-MP-F/R) have been added to the detailed PCR protocol in the same section.
2. **PCR-Positive Plant Counts:** The number of RT-PCR-positive individual plants for TPNRBV in each pool has been clearly stated in the Results section and is now also summarized in Supplementary Table 2.

These additions ensure that all critical parameters for the experimental verification of TPNRBV are fully disclosed, allowing for exact replication of our validation assays.

- *Results wording. The sentence “Low genetic variability between TPNRBV isolates based on the phylogenetic analysis indicates the absence of different isolates, and therefore the lack of a quasispecies structure.” over-interprets pooled data and conflicts with the reported SNVs.*

We thank the reviewer for this important comment. We agree that the cited sentence was an over-interpretation. We have removed it from the revised manuscript.

In the revised Results, we now simply state: *"Low genetic variability was observed between TPNRBV isolates based on the phylogenetic trees."*

The integration of this finding with the SNV data is now presented in the Discussion, where we suggest the low-frequency SNVs likely reflect a mixture of closely related field isolates rather than a within-host quasispecies.

- *Phylogeny reporting. Specify the number of comparator isolates per segment and list all accessions (country/year/host) in the main text or a Supplementary Table. If you use “Clade I/II,” define the labels and the criteria for assignment.*

**We thank the reviewer for the constructive suggestion to improve our phylogenetic reporting.**

**Clade Definition:** We have added a clear definition for 'Clade I' and 'Clade II' in the Figure 5 legend and the Results section. These labels refer to the two major, well-supported phylogenetic groups consistently recovered in our maximum likelihood analysis (performed with 1000 bootstrap replicates; nodes with support ≥50% are shown).

**Sequence Metadata and Selection Criteria:** In accordance with the reviewer's comment, we have updated Supplementary Table 3 to include both host plant and year of collection (where available) for each sequence used in the analysis. The table already contained the GenBank accession numbers, isolate designations, and country of origin. We emphasize that our phylogenetic analysis was intentionally based on complete genome sequences of all TPNRBV segments available in public databases to ensure a robust and congruent comparison.

These revisions provide full transparency regarding our methodology and the data used, facilitating the replication and interpretation of our phylogenetic findings."

Supplementary Table 3. Information on Tea plant necrotic ring blotch virus sequences used in phylogenetic analysis

| Isolate Location | Host | Year | GenBank accession number | | | |
| --- | --- | --- | --- | --- | --- | --- |
|  |  |  | RNA1 | RNA2 | RNA3 | RNA4 |
| TPNRBV_HZ China:  Hangzhou: Zhejiang  TPNRBV-WY6 China: Fujian | *Camellia sinensis*  *Camellia sinensis* | 2018  2023 | NC_040401, MG781152  OQ948455 | NC_040404, MG781153  OQ948425 | NC_040403, MG781154    OQ948435 | NC_040402, MG781155  OQ948445 |
| TPNRBV- W2 China: Fujian | *Camellia sinensis* | 2023 | OQ948456 | OQ948426 | OQ948436 | OQ948446 |
| TPNRBV-LJ30 China: Fujian | *Camellia sinensis* | 2023 | OQ948457 | OQ948427 | OQ948437 | OQ948447 |
| TPNRBV-LJ31 China: Fujian | *Camellia sinensis* | 2023 | OQ948458 | OQ948428 | OQ948438 | OQ948448 |
| TPNRBV-QZHA10 China: Fujian | *Camellia sinensis* | 2023 | OQ948459 | OQ948429 | OQ948439 | OQ948449 |
| TPNRBV-QZHA20 China: Fujian | *Camellia sinensis* | 2023 | OQ948460 | OQ948430 | OQ948440 | OQ948450 |
| TPNRBV-QZHA21 China: Fujian | *Camellia sinensis* | 2023 | OQ948461 | OQ948431 | OQ948441 | OQ948451 |
| TPNRBV-QZHA28 China: Fujian | *Camellia sinensis* | 2023 | OQ948462 | OQ948432 | OQ948442 | OQ948452 |
| TPNRBV-QZHA30 China: Fujian | *Camellia sinensis* | 2023 | OQ948463 | OQ948433 | OQ948443 | OQ948453 |
| TPNRBV-QZHA92 China: Fujian | *Camellia sinensis* | 2023 | OQ948464 | OQ948434 | OQ948444 | OQ948454 |
