# Supplementary material for: First complete genome sequences and transcript expression profiling of tea plant necrotic ring blotch virus isolates from Iran
Source: PLoS One. 2026 Aug 25;21(8):e0354634. doi: 10.1371/journal.pone.0354634 (PMC13505901; doi:10.1371/journal.pone.0354634)
Supplement: Peer review history

# Peer review: Decision Letter 0

**DOI:** 10.1371/journal.pone.0354634.r001

**Authors:** Surya Saha

26 Dec 2025

Dear Dr. Koolivand,

Please review excellent feedback provided that will improve this manuscript.

Please submit your revised manuscript by Feb 09 2026 11:59PM. If you will need more time than this to complete your revisions, please reply to this message or contact the journal office at plosone@plos.org. When you're ready to submit your revision, log on to https://www.editorialmanager.com/pone/ and select the 'Submissions Needing Revision' folder to locate your manuscript file.

Please include the following items when submitting your revised manuscript:

- A letter that responds to each point raised by the academic editor and reviewer(s). You should upload this letter as a separate file labeled 'Response to Reviewers'.
- A marked-up copy of your manuscript that highlights changes made to the original version. You should upload this as a separate file labeled 'Revised Manuscript with Track Changes'.
- An unmarked version of your revised paper without tracked changes. You should upload this as a separate file labeled 'Manuscript'.

If you would like to make changes to your financial disclosure, please include your updated statement in your cover letter. Guidelines for resubmitting your figure files are available below the reviewer comments at the end of this letter.

https://journals.plos.org/plosone/s/submission-guidelines#loc-laboratory-protocols. Additionally, PLOS ONE offers an option for publishing peer-reviewed Lab Protocol articles, which describe protocols hosted on protocols.io. Read more information on sharing protocols at https://plos.org/protocols?utm_medium=editorial-email&utm_source=authorletters&utm_campaign=protocols.

We look forward to receiving your revised manuscript.

Kind regards,

Surya Saha, PhD

Academic Editor

PLOS One

Journal Requirements:

When submitting your revision, we need you to address these additional requirements.

1. Please ensure that your manuscript meets PLOS ONE's style requirements, including those for file naming. The PLOS ONE style templates can be found at

https://journals.plos.org/plosone/s/file?id=wjVg/PLOSOne_formatting_sample_main_body.pdf and

https://journals.plos.org/plosone/s/file?id=ba62/PLOSOne_formatting_sample_title_authors_affiliations.pdf

2. In your Methods section, please provide additional information regarding the permits you obtained for the work. Please ensure you have included the full name of the authority that approved the field site access and, if no permits were required, a brief statement explaining why.

3. Your ethics statement should only appear in the Methods section of your manuscript. If your ethics statement is written in any section besides the Methods, please delete it from any other section.

4. Please ensure that you refer to Figures 1, 2 and 4 in your text as, if accepted, production will need this reference to link the reader to the figure.

5. Please include a copy of Tables Supplementary 1 and 2 which you refer to in your text on page 13.

6. If the reviewer comments include a recommendation to cite specific previously published works, please review and evaluate these publications to determine whether they are relevant and should be cited. There is no requirement to cite these works unless the editor has indicated otherwise.

7. Please review your reference list to ensure that it is complete and correct. If you have cited papers that have been retracted, please include the rationale for doing so in the manuscript text, or remove these references and replace them with relevant current references. Any changes to the reference list should be mentioned in the rebuttal letter that accompanies your revised manuscript. If you need to cite a retracted article, indicate the article’s retracted status in the References list and also include a citation and full reference for the retraction notice.

[Note: HTML markup is below. Please do not edit.]

Reviewers' comments:

Reviewer's Responses to Questions

**Comments to the Author**

1. Is the manuscript technically sound, and do the data support the conclusions?

Reviewer #1: Yes

Reviewer #2: Yes

Reviewer #3: Yes

**********

2. Has the statistical analysis been performed appropriately and rigorously?

Reviewer #1: Yes

Reviewer #2: Yes

Reviewer #3: Yes

**********

3. Have the authors made all data underlying the findings in their manuscript fully available?

The PLOS Data policy

Reviewer #1: Yes

Reviewer #2: Yes

Reviewer #3: Yes

**********

4. Is the manuscript presented in an intelligible fashion and written in standard English?

Reviewer #1: Yes

Reviewer #2: Yes

Reviewer #3: Yes

**********

Reviewer #1: The authors used the HTS method to obtain complete sequences of Tea Plant Necrotic Ring Blotch Virus (TPNRBV) from Iran. They also used HTS data for viral gene expression analysis and SNP analysis.

The paper is well written. The methods and procedures used are well described. The results are presented in considerable detail. The discussion summarizes the results achieved and compares them with previous work by other authors.

The two complete TPNRBV sequences obtained are the first complete sequences of this virus from Iran and the first complete sequences of this virus outside China and Japan. The significance of this work therefore lies in expanding knowledge about the genetic diversity and evolution of this virus, which has a major impact on tea production.

However, in order to publish this work, it is necessary to make adjustments to the formal processing of the manuscript and clarify the terminology.

The authors should mention their previous work on this virus from Scientific Reports (2023) and its connection to the current work. I see a considerable thematic similarity with this previously published work. As part of the review process, I do not have access to the supplementary files for the manuscript.

The images are missing numbers.

Line 42: The abstract repeats information about gene expression.

Lines 59-61: Blunervirus camelliae does not infect anything. It is a taxonomic construct. The name of the virus that infects tea plants is tea plant necrotic ring blotch virus.

Therefore, the sentence

"The disease is caused by Blunervirus camelliae (tea plant necrotic ring blotch virus (TPNRBV)), a member of the genus Blunervirus in the Kitaviridae family, first identified in China in 2018 through metagenomic sequencing."

should read as follows:

"The disease is caused by tea plant necrotic ring blotch virus (TPNRBV), species Blunervirus camelliae, a member of the genus Blunervirus in the Kitaviridae family, first identified in China in 2018 through metagenomic sequencing."

Lines 86, 255, 264: change NGS to HTS

Lines 188-189: similarity to other isolates – please specify and provide a link to images with sequence comparisons

Lines 191 (and 268-271): these are data from pooled samples, not from a single plant. Therefore, I would not use the term quasispecies, which refers to a virus in a single plant.

Line 216: RT-PCR does not confirm the presence of the virus in the dataset, but in the plant.

Line 219: The aim of sequencing RT-PCR products should be to confirm the sequences obtained using HTS, not to compare them with sequences in GenBank.

Lines 231-234: The description of the phylogenetic relationships of Iranian isolates does not correspond to the figure.

Lines 256-257: Better formulate the sentences to make it clear what was done in this work and what was done in the previous one. In addition, there is no reference to the previous work.

Lines 292-298: The information on gene expression is unnecessarily duplicated here. It would also be appropriate to mention the reason why the P22 and P14 genes are attributed the function of virus-host interaction and RNA silencing suppression, respectively.

Reviewer #2: This manuscript represents a high-quality piece of research that provides foundational resources and novel insights into TPNRBV. It adheres to sound scientific principles and its conclusions are well-supported by the data. The suggested revisions are minor and should be straightforward to address, after which the manuscript will be suitable for publication in PlOS ONE journal.

Lines 101-102: Beyond necrotic ring blotch and discoloration, what other specific "virus-like symptoms" were observed and recorded during the sample collection?

Line 103: The text refers to two pre-existing transcriptomic datasets [10]. Please give two transcriptomic datasets generation and references.

Line 104: Please add data regarding samples used for RT-PCR of both detected viruses (numbers, locations, symptoms…as well as their results in the Results section.)

Lines 109-110: About the library preparation, please write the type of RNA library which was constructed.

Lines 110-111: Concerning quality control: Beyond agarose gel electrophoresis, were any more sensitive methods (such as Bioanalyzer or Qubit) used to quantify RNA integrity (RIN) and concentration? Please write the specific quality thresholds for an RNA sample to be included in the pool.

Lines113-116: Regarding the host subtraction step, please, write the specific alignment parameters (e.g., percentage of identity, length fraction) which were used to map the reads to the (Camellia sinensis) genome (GCF_004153795.1), and the percentage of the total cleaned reads which were successfully mapped and removed as host-derived ones.

Lines 118-119: Please add more details about the BLAST filtering criteria. The section mentions filtering with "Open Blast Output results." What specific E-value and percentage identity thresholds were used for the BLASTn and BLASTx searches to define a significant viral hit? Adding these data would be more informative.

Line 118: For the TPNRBV contigs, what were the key BLAST statistics (E-value, percent identity, query coverage) that provided high confidence in its identification, and did the BLASTx analysis reveal any conserved domains in the assembled proteins? Please add the regarding data to the text.

Line 119: On the minimum contig length, what was the rationale behind choosing a 700 nucleotide (nt) minimum length for viral contigs? Wouldn't this filter potentially exclude smaller viral genes or genomes, and were any significant hits below this threshold observed and discarded?

Lines 132-134: Concerning the (de novo) assembly, please, write assembly algorithm and parameters (e.g., k-mer size, word size) which were used using the CLC Genomics Workbench, and parameters which were used for assessing the quality of the assembly (e.g., N50 contig length)

Lines 132-134: Regarding the assembly parameters, the length and similarity fractions for mapping were set to relatively low thresholds (0.5 and 0.8). What was the rationale for using these permissive parameters, and how did you ensure this did not lead to the misassembly of highly divergent or unrelated sequences?

Line 136: The minimum variant frequency was set to 1% (0.01), which is very sensitive. Given that the RNA was pooled from 20 different plants, how can you distinguish between a true low-frequency variant within one plant's viral population and a variant that is fixed but only present in a small subset of the infected plants in the pool? Please consider such limitations in conclusion.

Line 137: Beyond the statistical p-value, were any of the identified non-synonymous SNPs, especially in key genes like P22, validated by an independent method like Sanger sequencing to rule out potential sequencing or mapping artifacts? Add them to the text.

Lines 140-141: The SNPs were visualized on 3D protein structures. For the non-synonymous SNPs identified, did their locations on the 3D structure suggest a potential functional impact (e.g., were they located in active sites, binding interfaces, or stable domains)? What was the key structural insight gained? Please discuss these results in the manuscript.

Lines 175-176: On the sequencing output, please, add the total sequencing output (e.g., total gigabytes of data or number of raw reads generated) for each of the pooled libraries.

Lines 175-177: The 2022 library generated nearly 15 times more total contigs than the 2021 library (172,521 vs 11,638), despite having only about 2.5 times more clean reads. What factors account for this massive difference in assembly complexity? Could it be due to a higher diversity of other microorganisms (e.g., fungi, bacteria) in the 2022 samples, or different assembly parameters? Please discuss these results in the text.

Lines 179-180: The text states the (majority) of contigs were associated with TPNRBV. This implies a significant number of non-TPNRBV contigs were also assembled. What were the other top BLASTn hits for these non-TPNRBV contigs? Were any other novel or known plant viruses identified in these libraries? Please add these data and their discussion.

Lines 180-186: The TPNRBV genome has four segments. Were the coverage and depth of reads uniform across all four reference segments (RNA1-RNA4) during the mapping process, or were there regions with significantly lower coverage that might have been missed in the assembly and SNP analysis? Please add relates results to the text.

Lines 180-182: Although the total number of contigs differed greatly between libraries, the number of TPNRBV-related contigs was similar (35 vs 30). What was the average length and depth of coverage of these viral contigs in each library? Did the 2022 library, with more data, produce more complete or higher-quality TPNRBV contigs? Please discuss them in the text.

Lines 191-195: Considering the experimental design: Given that the samples were pooled, how can the study confidently attribute specific genetic variations (like the SNPs mentioned in the abstract) to individual viral isolates, rather than treating them as a mixture from a viral population?

Lines 193-196: The identified non-synonymous SNPs have a very broad frequency range (0.26% to 21.47%). How are these SNPs distributed across this range? Are most SNPs at very low frequency (e.g., below 2%), suggesting they are transient mutations, or is there a significant number at higher frequencies, suggesting they may be selectively advantageous? It would be more informative adding these data to the results and discussion sections.

Lines 198-201: For the non-synonymous SNPs found at the highest frequencies (e.g., >10%), did in silico protein analysis (like SIFT or PROVEAN) predict whether these amino acid changes would be deleterious, neutral, or beneficial to the protein's function? It would be more informative adding these data to the results and discussion sections.

Lines 199-201: RNA1 and RNA2 harbor the vast majority (81 out of 93) of the non-synonymous SNPs. Does the high mutation load correlate with known variable domains (e.g., the RNA-dependent RNA polymerase in RNA1), suggesting potential escape mutants or adaptability? Please enter it to the discussion.

Lines 200-201: The 15 SNPs shared between the 2021 and 2022 collections are highly interesting. What are the specific amino acid changes and which proteins are affected by these shared, conserved mutations? Does their conservation across seasons suggest they confer a selective advantage in the Iranian tea plant environment? Results and discussion on this topic in the manuscript would be informative and interesting.

Lines 203-204: The analysis showed "no visible changes" in the 3D structure. What was the resolution of this analysis? Was it a simple visual inspection, or were quantitative metrics (e.g., changes in binding pocket volume, electrostatic surface potential, or protein stability) calculated? Could the mutations affect properties not visible in a static structure, like protein flexibility or interaction networks? Adding these data will contribute to the scientific content of the manuscript.

Lines 206-209: Regarding expression normalization: The study uses TPM for normalization. Was the "total mapped reads" used in the TPM denominator the total number of reads that mapped specifically to the TPNRBV genome, or was it the total number of reads in the entire sequencing library (including plant and other microbial reads)?

Lines 229-233: The phylogenetic trees for the different RNA segments were discordant (RNA1 vs. RNA2-4). The discussion states a close relationship to Hangzhou isolates but doesn't address this mixed signal. How do you interpret this conflicting phylogenetic history in the absence of more detectable recombination? Also, the discordant phylogenetic trees for RNA1 (groups with Fujian) versus RNA2-RNA4 (groups with Hangzhou) is a critical finding that is buried in the results and not adequately discussed. The discussion should explicitly address this and hypothesize about its cause, especially since recombination was weak. It would be better to mention these issues in the discussion.

Lines 245-248: The Iranian isolates share a pattern of conserved 5' terminal motifs with the Hangzhou isolate (absent in RNA2-RNA4, a specific variant in RNA1). Do you have data about the critical role of these motifs for viral replication? Does their shared, atypical structure provide stronger evidence for a direct evolutionary link than the nucleotide identity percentages? Enter this data for clarifying importance of the conserved terminal motifs.

Lines 250-253: The 110-nt insertion in RNA4 is a unique marker shared only by the Iranian and Hangzhou isolates. Does this insertion occur in a coding or non-coding region? What is its predicted functional impact, and does its presence override the phylogenetic signal from RNA1, making a Hangzhou origin more likely for the majority of the genome? This discussion would add the significance of the RNA4 insertion.

Lines 257-260: The study concludes the mapped reads were sufficient for full genome coverage. What was the minimum depth of coverage across the entire TPNRBV genome in each library, and were there any regions with consistently low coverage that might be prone to assembly errors?

Lines 267-271: The SNP data indicates a diverse quasispecies, while the phylogenetic trees show low variability. Given that the pooling strategy is the most likely cause, what specific follow-up experiment did you use to definitively resolve whether this diversity represents a true quasispecies within single plants or a mixture of very similar but distinct isolates across the plantation? Please clear this Quasispecies issue.

Lines 272-273: Were the 93 non-synonymous SNPs evenly distributed across the four segments of the TPNRBV genome (RNA1-RNA4), or were they concentrated in specific segments or genes (e.g., the highly expressed P22 gene)? What might a cluster of mutations in one particular gene suggest? Please add data regarding these questions to the results and discussion sections.

Lines 290-291: For the highly expressed P22 gene, what was the average read depth of coverage across its ORF? Was the read coverage uniform, or were there regions with unusually high or low coverage that might suggest post-transcriptional processing or assembly artifacts? Please add and discuss related results.

Lines 304-305: The statement that P22 has "no SNPs" should be nuanced. It would be more accurate to state that “no SNPs were found (above the 1% frequency threshold in this particular sample pool)”. This leaves open the possibility of lower-frequency variants or variation in other populations.

Lines 306-312: The Iranian isolates group with Fujian in the RNA1 tree but with Hangzhou in the RNA2-RNA4 trees. If RDP4 detected no recombination, what other evolutionary mechanisms could explain this apparent reassortment of genomic segments? Does this suggest the Iranian isolates could be a natural reassortant between two different Chinese TPNRBV lineages? Please enter these points in the discussion which will contribute to the article’s appeal and scientific clarity.

Lines 306-308: Given the high similarity to Chinese isolates, the mixed phylogeny, and the shared unique insertion, what is the most plausible hypothesis for the emergence of TPNRBV in Iran? Does the evidence point more strongly to a single introduction event from a population with mixed ancestry, or multiple independent introductions of different segments? It is recommended that this issue be discussed by including more data in addition to presenting the phylogeny trees.

Reviewer #3: The manuscript titled “First Complete Genome Sequences and Transcript Expression Profiling of Tea Plant Necrotic Ring Blotch Virus Isolates from Iran” by Esmaeilzadeh et al., report the first complete genome sequences of two Tea plant necrotic ring blotch virus (TPNRBV) isolates from Iran and analyze ORF expression (TPM) and within-host variation (SNPs) using pooled RNA-seq from 2021 and 2022. They show close relatedness to Chinese isolates, identify a 110-nt insertion in RNA4, and note high expression of P22. The work addresses a real gap in Iranian TPNRBV genomics and is relevant for tea pathology and regional surveillance.

Major strengths

• First complete TPNRBV genomes from Iran with segment-wise phylogenies (RNA1–RNA4).

• A coherent analysis package—assembly, expression, SNP calling, recombination, and phylogeny—from existing RNA-seq data.

• Clear practical motivation for disease monitoring and diagnostics in tea.

Points to improve (readability and rigor)

• Taxonomy/nomenclature. The species and common names are mixed (“Blunervirus camelliae (TPNRBV)”). Please standardize usage across the manuscript: species = Blunervirus camelliae; virus/common name = TPNRBV. Pick one convention and apply it consistently.

• Vector/transmission statements. The text mentions mechanical and seed transmission and hypothesized insect vectors (mites, aphids, leafhoppers, whiteflies). Please align these statements with current evidence for kitavirids/blunerviruses (e.g., Brevipalpus mites for related viruses) and cite primary sources. If evidence is lacking for TPNRBV, say so explicitly.

• Pooled samples and interpretation. Because samples were pooled by year, allele frequencies reflect mixtures of infections across plants, not clonal within-host dynamics. Likewise, TPM differences from pooled RNA-seq cannot be interpreted as within-host expression changes. Please reframe conclusions accordingly (no linkage inference; population-level, not within-host, signals).

• Phylogeny reporting. Specify the number of comparator isolates per segment and list all accessions (country/year/host) in the main text or a Supplementary Table. If you use “Clade I/II,” define the labels and the criteria for assignment.

• PCR verification details. Provide primer sequences (TPNRBV3-F/R, MP-F/R), exact annealing temperatures (rather than “respective temperatures”), and the counts of PCR-positive plants per pool. This will improve reproducibility.

• Results wording. The sentence “Low genetic variability between TPNRBV isolates based on the phylogenetic analysis indicates the absence of different isolates, and therefore the lack of a quasispecies structure.” over-interprets pooled data and conflicts with the reported SNPs.

Overall assessment. The manuscript is scientifically sound and generally well supported by relevant references. It integrates viral genome sequencing, expression profiling, SNP discovery, recombination screening, and segment-wise phylogeny for TPNRBV—highly relevant to tea pathology and surveillance—making this a potentially valuable contribution to the field. With careful revision along the points above, the work will be significantly stronger and its central claims clearer and it is highly recommended for publication after minor revision.

**********

what does this mean?). If published, this will include your full peer review and any attached files.

If you choose “no”, your identity will remain anonymous but your review may still be made public.

**Do you want your identity to be public for this peer review?**  For information about this choice, including consent withdrawal, please see our Privacy Policy

Reviewer #1: No

Reviewer #2: No

Reviewer #3: No

**********

[NOTE: If reviewer comments were submitted as an attachment file, they will be attached to this email and accessible via the submission site. Please log into your account, locate the manuscript record, and check for the action link "View Attachments". If this link does not appear, there are no attachment files.]

To ensure your figures meet our technical requirements, please review our figure guidelines: https://journals.plos.org/plosone/s/figures

You may also use PLOS’s free figure tool, NAAS, to help you prepare publication quality figures: https://journals.plos.org/plosone/s/figures#loc-tools-for-figure-preparation.

NAAS will assess whether your figures meet our technical requirements by comparing each figure against our figure specifications.

# Author comment: Author response to Decision Letter 1

**DOI:** 10.1371/journal.pone.0354634.r002

9 Feb 2026

Dear Editor,

Thank you for your valuable reviewers’ comments. Your feedback has significantly contributed to the enhancement of our manuscript, and we have addressed all the points raised by reviewers 1, 2, and 3. Please don't hesitate to share any additional comments or questions you may have; we are committed to further improving our manuscript. The changes have been meticulously documented using the different color feature in Microsoft Word, and we have provided detailed responses to each of the reviewer's comments.

Reviewer #1

The authors should mention their previous work on this virus from Scientific Reports (2023) and its connection to the current work. I see a considerable thematic similarity with this previously published work. As part of the review process, I do not have access to the supplementary files for the manuscript.

We thank the reviewer for raising this important point. To clearly establish the connection between the previous and current studies, we have added the following paragraph at the end of the Background section:

“This study builds directly upon our previous work (Esmaeilzadeh et al., 2023), which reported the first molecular and biological characterization of TPNRBV in Iran. While that study confirmed the presence of the virus and described its basic biological properties, the present research applies high-throughput sequencing to generate the first complete genome sequences of Iranian TPNRBV isolates. This approach enables comprehensive genomic, phylogenetic, and population-level analyses. Together, these studies provide an integrated and detailed characterization of TPNRBV in Iran.”

This addition clarifies the logical progression from our initial discovery and characterization of TPNRBV to the current in-depth genomic investigation and highlights the novel contributions of the present manuscript.

Reviewer #1

The images are missing numbers

Thank you for your comment. The figure numbers have now been added to the images as requested

lines 42:

The abstract repeats information about gene expression

Thank you for your valuable comment. As suggested, the redundant sentence concerning transcript expression analysis has been removed from the Abstract to avoid repetition.

lines 59-61:

Blunervirus camelliae does not infect anything. It is a taxonomic construct. The name of the virus that infects tea plants is tea plant necrotic ring blotch virus.

Therefore, the sentence

"The disease is caused by Blunervirus camelliae (tea plant necrotic ring blotch virus (TPNRBV)), a member of the genus Blunervirus in the Kitaviridae family, first identified in China in 2018 through metagenomic sequencing."

should read as follows:

The sentence was revised according to the reviewer's comment as follows: The disease is caused by tea plant necrotic ring blotch virus (TPNRBV), species Blunervirus camelliae, a member of the genus Blunervirus in the Kitaviridae family, first identified in China in 2018 through metagenomic sequencing

line 216:

RT-PCR does not confirm the presence of the virus in the dataset, but in the plant.

The sentence was revised according to the reviewer's comment as follows: RT-PCR confirmed the presence of TPB-Iran and TPNRBV-Ir in the individual samples used for the pools using two specific primer pairs for TPNRBV

line 219:

The aim of sequencing RT-PCR products should be to confirm the sequences obtained using HTS, not to compare them with sequences in GenBank.

The sentence was revised according to the reviewer's comment as follows: BLASTn analysis of the resulting fragments confirmed the presence of TPNRBV in these plants.

lines 86, 255, 264:

change NGS to HTS

NGS was changed to HTS.

lines 188-189:

similarity to other isolates – please specify and provide a link to images with sequence comparisons

We thank the reviewer for this suggestion. We have updated the text to specify the similarity. The pairwise nucleotide identity between our Iranian isolates (TPB-Iran/TPNRBV-Ir) and the closest isolates in GenBank is as follows:

● RNA1: 96.13% identity with the TPNRBV Fujian isolate.

● RNA2: 96.38% identity with the TPNRBV Hangzhou isolate (NC_040402).

● RNA3: 97.27% identity with the TPNRBV Hangzhou isolate (NC_040403).

● RNA4: 94.89% identity with the TPNRBV Hangzhou isolate (NC_040404).

As requested, we provide a visual comparison in the form of a pairwise identity matrix, which is presented in Fig. 5. This figure allows for easy comparison of sequence similarity across all isolates analyzed in this study.

lines 231-234:

The description of the phylogenetic relationships of Iranian isolates does not correspond to the figure

We thank the reviewer for this comment. To eliminate any ambiguity, we have now added clear labels (Clade I and Clade II) to the relevant branches in Figure 5. The description in the text (The two major phylogenetic groups in the trees were designated Clade I and Clade II. These clades represent well-supported and consistent divisions, with bootstrap values ≥ 50% at the defining nodes (Fig. 5)) accurately corresponds to the labeled topology in the revised figure

lines 191 (and 268-271):

these are data from pooled samples, not from a single plant. Therefore, I would not use the term quasispecies, which refers to a virus in a single plant.

We thank the reviewer for this crucial comment. We fully agree. The term "quasispecies" has been removed throughout the manuscript (including in the Abstract, Results, and Discussion sections) and replaced with more accurate descriptors such as "genetic diversity within the viral population" and "intra-population sequence variation" to reflect that our data derive from pooled samples representing a field population

Reviewer #2

lines 101-102:

Beyond necrotic ring blotch and discoloration, what other specific "virus-like symptoms" were observed and recorded during the sample collection?

Thank you for your question. Other specific symptoms were indeed observed during sample collection. We have clarified this in the 'Sample collection' subsection. The added text is:

"...including necrotic ring blotch, discoloration (albino and chlorine), yellowing, ring spots, and mosaic."

line 103:

The text refers to two pre-existing transcriptomic datasets [10]. Please give two transcriptomic datasets generation and references.

We thank the reviewer for this suggestion. We have now clarified the origin of the two transcriptomic datasets in the Materials and Methods section. The added text reads: "This study is based on integrated analyses of two original transcriptomic datasets generated by our group. The first dataset was produced from an RNA-seq experiment conducted in 2021, and the second from a separate RNA-seq experiment conducted in 2022. The methodology for library preparation and sequencing was consistent across both projects and is detailed herein.

line 104:

Please add data regarding samples used for RT-PCR of both detected viruses (numbers, locations, symptoms…as well as their results in the Results section)

We thank the reviewer for highlighting the importance of these validation data. All requested information is included in the table below:

Table 2. Summary of sample collection and RT-PCR detection results for TPNRBV and CoAV1, aggregated by major geographical region.

Region (Province) No of Samples Predominant Symptoms TPNRBV-Positive CoAV-Positive Co-infection

(TPNRBV+CoAV1)

Guilan Province

Mazandaran Province

Total

12

8

20

necrotic ring blotch

discoloration (albino and chlorine)

yellowing,

ring spots

mosaic

11

8

19

3

2

5

3

1

4

We believe these data, presented in the indicated sections, provide a clear and complete account of the sample set and validation results."

lines 109-110:

About the library preparation, please write the type of RNA library which was constructed.

RNA-Seq libraries were then prepared using the TruSeq Stranded Total RNA kit according to the manufacturer's instructions. The libraries were sequenced by Novogene (China) on an Illumina HiSeq 2000 platform, generating 150 bp paired-end reads.

lines 110-111:

Concerning quality control: Beyond agarose gel electrophoresis, were any more sensitive methods (such as Bioanalyzer or Qubit) used to quantify RNA integrity (RIN) and concentration? Please write the specific quality thresholds for an RNA sample to be included in the pool.

The quality and concentration of the extracted RNA were assessed using agarose gel electrophoresis and Nanodrop spectrophotometer (Thermo Fisher Scientific, USA). Equal amounts of RNA from each sample were pooled. Integrity and purity of the total RNA were also analyzed using a Bioanalyzer 2100 based on an RIN number > 7. RNA-Seq libraries were then prepared using the TruSeq Stranded Total RNA Preparation Kit with Ribo-Zero depletion according to the manufacturer's instructions. The libraries were sequenced by Novogene (China) on an Illumina HiSeq 2000 platform, generating 150 bp paired-end reads.

lines 113-116:

Regarding the host subtraction step, please, write the specific alignment parameters (e.g., percentage of identity, length fraction) which were used to map the reads to the (Camellia sinensis) genome (GCF_004153795.1), and the percentage of the total cleaned reads which were successfully mapped and removed as host-derived ones.

The cleaned and trimmed reads were then mapped to the tea genome (Camellia sinensis genome GCF_004153795.1_AHAU_CSS) using CLC Genomics Workbench 20 with default parameters and a length fraction and similarity fraction set to 0.8. From the clean reads, 27,244,799 and 78,269,958 reads were mapped to the tea genome.

lines 118-119:

Please add more details about the BLAST filtering criteria. The section mentions filtering with "Open Blast Output results." What specific E-value and percentage identity thresholds were used for the BLASTn and BLASTx searches to define a significant viral hit? Adding these data would be more informative.

We thank the reviewer for this insightful comment. To provide full clarity, we have revised the relevant section in the manuscript. Our filtering criteria involved the following steps:

1. Bioinformatics Screening: Contigs with a Greatest HSP Length ≥ 700 nucleotides in the BLASTn results (using an E-value cutoff of 1e-5) were selected as candidates. Their nucleotide identity to known viruses was high (e.g., >84% for TPNRBV).

2. Experimental Verification: The viruses reported in this study were definitively identified and confirmed using virus-specific RT-PCR assays, followed by Sanger sequencing. This verification is detailed in the 'Verification of HTS data' section.

Furthermore, an unrestricted BLASTn search of all contigs was conducted to detect very small agents like viroids, with no significant hits found.

These revisions now clearly reflect our selection criteria and confirmatory path.

line 118:

For the TPNRBV contigs, what were the key BLAST statistics (E-value, percent identity, query coverage) that provided high confidence in its identification, and did the BLASTx analysis reveal any conserved domains in the assembled proteins? Please add the regarding data to the text.

We thank the reviewer for this suggestion. The key BLAST statistics and conserved domain information are already provided in the Results section of our manuscript. For the reviewer's convenience, we reiterate the data here:

- BLASTn analysis of the assembled contigs revealed high-confidence matches (E-value = 0.0, Query Coverage = 100%) with the following percent identities for each segment: RNA1: 96.13% to a Fujian isolate (OQ948458); RNA2: 96.38%; RNA3: 97.27%; and RNA4: 94.89% to Hangzhou isolates (NC_040402-NC_040404).

Genomic Segment Closest Isolate (Accession) E-value Percent Identity Query Coverage

RNA1 Fujian (OQ948458) 0.0 96.13% 100%

RNA2 Hangzhou (NC_040402) 0.0 96.38% 100%

RNA3 Hangzhou (NC_040403) 0.0 97.27% 100%

RNA4 Hangzhou (NC_040404) 0.0 94.89% 100%

- Furthermore, BLASTx analysis confirmed the presence of conserved domains characteristic of TPNRBV, including methyltransferase-helicase (RNA1), helicase-RNA polymerase (RNA2), a virion membrane protein (RNA3-P24), and a movement protein (RNA4).

line 119:

On the minimum contig length, what was the rationale behind choosing a 700 nucleotide (nt) minimum length for viral contigs? Wouldn't this filter potentially exclude smaller viral genes or genomes, and were any significant hits below this threshold observed and discarded?

We thank the reviewer for raising this critical point regarding the potential exclusion of small viral agents.

The 700 nt threshold was employed as a conservative filter to prioritize high-confidence contigs for de novo assembly and analysis of conventional plant viruses. This heuristic is common in virome studies to reduce false positives from short, non-specific matches.

We fully agree with the reviewer that this filter would, in principle, exclude viroids and very small viral satellites. Therefore, to ensure a comprehensive survey, we performed an additional, dedicated analysis. Specifically, we conducted a separate BLASTn search of all assembled contigs and unassembled reads against the entire viral RefSeq database, including viroid sequences.

The results of this sensitive, unrestricted search confirmed that no significant hits (E-value < 1e-5) to viroids or other small viral agents were present in our samples. All significant viral signals corresponded to the two viruses we report: Tea plant necrotic ring blotch virus (TPNRBV) and Citrus-associated arlivirus 1 (CoAV1). Both of these viruses assembled into contigs well above our 700 nt cutoff (complete segments for TPNRBV and substantial contigs for CoAV1).

Thus, while the 700 nt filter served as a practical step for streamlining the focus on larger viruses, it did not lead to the loss of any biologically relevant small agents in this specific dataset. Our complementary unbiased screening validated the absence of viroids and confirmed that the viral community was dominated by TPNRBV and CoAV1, both successfully captured by our assembly pipeline. We have clarified this two-tiered analytical approach in the revised Methods section.

lines 132-134:

Concerning the (de novo) assembly, please, write assembly algorithm and parameters (e.g., k-mer size, word size) which were used using the CLC Genomics Workbench, and parameters which were used for assessing the quality of the assembly (e.g., N50 contig length)

Thank you for the comment. The de novo assembly was performed using the "De Novo Assembly" tool in CLC Genomics Workbench v20. The key parameters set for the assembly were: word size (for read overlap)= 20 and minimum contig length = 200 bp. The CLC assembler uses a proprietary algorithm, and as it is not a k-mer-based assembler, a k-mer size parameter was not applicable in this workflow.

The quality of the assembled viral contigs was assessed based on the following criteria: Contig Length & Coverage: All viral contigs were >700 bp and provided contiguous, full-length coverage of the four reference genome segments (RNA1-RNA4). High Sequencing Depth: The read coverage across all segments was deep and uniform (see Fig. 2a, b), with no regions below the minimum coverage threshold. Experimental Validation: The assembled sequences were validated by RT-PCR and Sanger sequencing of key genomic regions.

We note that the N50 statistic, commonly reported for de novo assembly of a complete transcriptome, was not a primary focus of our analysis. Our goal was specifically to assemble and validate the viral genomes from the complex plant transcriptome data. The most direct and relevant quality metrics for this viral-focused aim are the three criteria detailed above (contig completeness, depth, and experimental validation).

We will revise the Materials and Methods section to include the specific assembly parameters mentioned.

lines 132-134:

Regarding the assembly parameters, the length and similarity fractions for mapping were set to relatively low thresholds (0.5 and 0.8). What was the rationale for using these permissive parameters, and how did you e

Attachment Submitted filename: Response to reviewer Final 15-F.docx

# Peer review: Decision Letter 1

**DOI:** 10.1371/journal.pone.0354634.r003

**Authors:** Murtada D. Naser

9 Jul 2026

Dear Dr. Davoud Koolivand,

Thank you for submitting your manuscript to PLOS ONE. After careful consideration, we feel that it has merit but does not fully meet PLOS ONE’s publication criteria as it currently stands. Therefore, we invite you to submit a revised version of the manuscript that addresses the points raised during the review process.

Please note the reviewers comments about connecting this to previous work. Most of the suggested revisions are minor so I hope that we can get a response quickly.

Please submit your revised manuscript by Feb 28 2026 11:59PM. If you will need more time than this to complete your revisions, please reply to this message or contact the journal office at plosone@plos.org. When you're ready to submit your revision, log on to https://www.editorialmanager.com/pone/ and select the 'Submissions Needing Revision' folder to locate your manuscript file.

- A letter that responds to each point raised by the academic editor and reviewer(s). You should upload this letter as a separate file labeled 'Response to Reviewers'.
- A marked-up copy of your manuscript that highlights changes made to the original version. You should upload this as a separate file labeled 'Revised Manuscript with Track Changes'.
- An unmarked version of your revised paper without tracked changes. You should upload this as a separate file labeled 'Manuscript'.

If applicable, we recommend that you deposit your laboratory protocols in protocols.io to enhance the reproducibility of your results. Protocols.io assigns your protocol its own identifier (DOI) so that it can be cited independently in the future. For instructions see: https://journals.plos.org/plosone/s/submission-guidelines#loc-laboratory-protocols. Additionally, PLOS ONE offers an option for publishing peer-reviewed Lab Protocol articles, which describe protocols hosted on protocols.io. Read more information on sharing protocols at https://plos.org/protocols?utm_medium=editorial-email&utm_source=authorletters&utm_campaign=protocols.

We look forward to receiving your revised manuscript.

Kind regards,

Surya Saha, PhD

Academic Editor

PLOS One

Journal Requirements:

If the reviewer comments include a recommendation to cite specific previously published works, please review and evaluate these publications to determine whether they are relevant and should be cited. There is no requirement to cite these works unless the editor has indicated otherwise.

Please review your reference list to ensure that it is complete and correct. If you have cited papers that have been retracted, please include the rationale for doing so in the manuscript text, or remove these references and replace them with relevant current references. Any changes to the reference list should be mentioned in the rebuttal letter that accompanies your revised manuscript. If you need to cite a retracted article, indicate the article’s retracted status in the References list and also include a citation and full reference for the retraction notice.

Additional Editor Comments (if provided):

Please note the reviewers comments about connecting this to previous work. Most of the suggested revisions are minor so I hope that we can get a response quickly from you.

[Note: HTML markup is below. Please do not edit.]

Reviewers' comments:

Reviewer 1

The authors used the HTS method to obtain complete sequences of Tea Plant Necrotic Ring Blotch Virus (TPNRBV) from Iran. They also used HTS data for viral gene expression analysis and SNP analysis.

The paper is well written. The methods and procedures used are well described. The results are presented in considerable detail. The discussion summarizes the results achieved and compares them with previous work by other authors.

The two complete TPNRBV sequences obtained are the first complete sequences of this virus from Iran and the first complete sequences of this virus outside China and Japan. The significance of this work therefore lies in expanding knowledge about the genetic diversity and evolution of this virus, which has a major impact on tea production.

However, in order to publish this work, it is necessary to make adjustments to the formal processing of the manuscript and clarify the terminology.

The authors should mention their previous work on this virus from Scientific Reports (2023) and its connection to the current work. I see a considerable thematic similarity with this previously published work. As part of the review process, I do not have access to the supplementary files for the manuscript.

The images are missing numbers.

Line 42: The abstract repeats information about gene expression.

Lines 59-61: Blunervirus camelliae does not infect anything. It is a taxonomic construct. The name of the virus that infects tea plants is tea plant necrotic ring blotch virus.

Therefore, the sentence

"The disease is caused by Blunervirus camelliae (tea plant necrotic ring blotch virus (TPNRBV)), a member of the genus Blunervirus in the Kitaviridae family, first identified in China in 2018 through metagenomic sequencing."

should read as follows:

"The disease is caused by tea plant necrotic ring blotch virus (TPNRBV), species Blunervirus camelliae, a member of the genus Blunervirus in the Kitaviridae family, first identified in China in 2018 through metagenomic sequencing."

Lines 86, 255, 264: change NGS to HTS

Lines 188-189: similarity to other isolates – please specify and provide a link to images with sequence comparisons

Lines 191 (and 268-271): these are data from pooled samples, not from a single plant. Therefore, I would not use the term quasispecies, which refers to a virus in a single plant.

Line 216: RT-PCR does not confirm the presence of the virus in the dataset, but in the plant.

Line 219: The aim of sequencing RT-PCR products should be to confirm the sequences obtained using HTS, not to compare them with sequences in GenBank.

Lines 231-234: The description of the phylogenetic relationships of Iranian isolates does not correspond to the figure.

Lines 256-257: Better formulate the sentences to make it clear what was done in this work and what was done in the previous one. In addition, there is no reference to the previous work.

Lines 292-298: The information on gene expression is unnecessarily duplicated here. It would also be appropriate to mention the reason why the P22 and P14 genes are attributed the function of virus-host interaction and RNA silencing suppression, respectively.

Reviewer 2

This manuscript represents a high-quality piece of research that provides foundational resources and novel insights into TPNRBV. It adheres to sound scientific principles and its conclusions are well-supported by the data. The suggested revisions are minor and should be straightforward to address, after which the manuscript will be suitable for publication in PlOS ONE journal.

Lines 101-102: Beyond necrotic ring blotch and discoloration, what other specific "virus-like symptoms" were observed and recorded during the sample collection?

Line 103: The text refers to two pre-existing transcriptomic datasets [10]. Please give two transcriptomic datasets generation and references.

Line 104: Please add data regarding samples used for RT-PCR of both detected viruses (numbers, locations, symptoms…as well as their results in the Results section.)

Lines 109-110: About the library preparation, please write the type of RNA library which was constructed.

Lines 110-111: Concerning quality control: Beyond agarose gel electrophoresis, were any more sensitive methods (such as Bioanalyzer or Qubit) used to quantify RNA integrity (RIN) and concentration? Please write the specific quality thresholds for an RNA sample to be included in the pool.

Lines113-116: Regarding the host subtraction step, please, write the specific alignment parameters (e.g., percentage of identity, length fraction) which were used to map the reads to the (Camellia sinensis) genome (GCF_004153795.1), and the percentage of the total cleaned reads which were successfully mapped and removed as host-derived ones.

Lines 118-119: Please add more details about the BLAST filtering criteria. The section mentions filtering with "Open Blast Output results." What specific E-value and percentage identity thresholds were used for the BLASTn and BLASTx searches to define a significant viral hit? Adding these data would be more informative.

Line 118: For the TPNRBV contigs, what were the key BLAST statistics (E-value, percent identity, query coverage) that provided high confidence in its identification, and did the BLASTx analysis reveal any conserved domains in the assembled proteins? Please add the regarding data to the text.

Line 119: On the minimum contig length, what was the rationale behind choosing a 700 nucleotide (nt) minimum length for viral contigs? Wouldn't this filter potentially exclude smaller viral genes or genomes, and were any significant hits below this threshold observed and discarded?

Lines 132-134: Concerning the (de novo) assembly, please, write assembly algorithm and parameters (e.g., k-mer size, word size) which were used using the CLC Genomics Workbench, and parameters which were used for assessing the quality of the assembly (e.g., N50 contig length)

Lines 132-134: Regarding the assembly parameters, the length and similarity fractions for mapping were set to relatively low thresholds (0.5 and 0.8). What was the rationale for using these permissive parameters, and how did you ensure this did not lead to the misassembly of highly divergent or unrelated sequences?

Line 136: The minimum variant frequency was set to 1% (0.01), which is very sensitive. Given that the RNA was pooled from 20 different plants, how can you distinguish between a true low-frequency variant within one plant's viral population and a variant that is fixed but only present in a small subset of the infected plants in the pool? Please consider such limitations in conclusion.

Line 137: Beyond the statistical p-value, were any of the identified non-synonymous SNPs, especially in key genes like P22, validated by an independent method like Sanger sequencing to rule out potential sequencing or mapping artifacts? Add them to the text.

Lines 140-141: The SNPs were visualized on 3D protein structures. For the non-synonymous SNPs identified, did their locations on the 3D structure suggest a potential functional impact (e.g., were they located in active sites, binding interfaces, or stable domains)? What was the key structural insight gained? Please discuss these results in the manuscript.

Lines 175-176: On the sequencing output, please, add the total sequencing output (e.g., total gigabytes of data or number of raw reads generated) for each of the pooled libraries.

Lines 175-177: The 2022 library generated nearly 15 times more total contigs than the 2021 library (172,521 vs 11,638), despite having only about 2.5 times more clean reads. What factors account for this massive difference in assembly complexity? Could it be due to a higher diversity of other microorganisms (e.g., fungi, bacteria) in the 2022 samples, or different assembly parameters? Please discuss these results in the text.

Lines 179-180: The text states the (majority) of contigs were associated with TPNRBV. This implies a significant number of non-TPNRBV contigs were also assembled. What were the other top BLASTn hits for these non-TPNRBV contigs? Were any other novel or known plant viruses identified in these libraries? Please add these data and their discussion.

Lines 180-186: The TPNRBV genome has four segments. Were the coverage and depth of reads uniform across all four reference segments (RNA1-RNA4) during the mapping process, or were there regions with significantly lower coverage that might have been missed in the assembly and SNP analysis? Please add relates results to the text.

Lines 180-182: Although the total number of contigs differed greatly between libraries, the number of TPNRBV-related contigs was similar (35 vs 30). What was the average length and depth of coverage of these viral contigs in each library? Did the 2022 library, with more data, produce more complete or higher-quality TPNRBV contigs? Please discuss them in the text.

Lines 191-195: Considering the experimental design: Given that the samples were pooled, how can the study confidently attribute specific genetic variations (like the SNPs mentioned in the abstract) to individual viral isolates, rather than treating them as a mixture from a viral population?

Lines 193-196: The identified non-synonymous SNPs have a very broad frequency range (0.26% to 21.47%). How are these SNPs distributed across this range? Are most SNPs at very low frequency (e.g., below 2%), suggesting they are transient mutations, or is there a significant number at higher frequencies, suggesting they may be selectively advantageous? It would be more informative adding these data to the results and discussion sections.

Lines 198-201: For the non-synonymous SNPs found at the highest frequencies (e.g., >10%), did in silico protein analysis (like SIFT or PROVEAN) predict whether these amino acid changes would be deleterious, neutral, or beneficial to the protein's function? It would be more informative adding these data to the results and discussion sections.

Lines 199-201: RNA1 and RNA2 harbor the vast majority (81 out of 93) of the non-synonymous SNPs. Does the high mutation load correlate with known variable domains (e.g., the RNA-dependent RNA polymerase in RNA1), suggesting potential escape mutants or adaptability? Please enter it to the discussion.

Lines 200-201: The 15 SNPs shared between the 2021 and 2022 collections are highly interesting. What are the specific amino acid changes and which proteins are affected by these shared, conserved mutations? Does their conservation across seasons suggest they confer a selective advantage in the Iranian tea plant environment? Results and discussion on this topic in the manuscript would be informative and interesting.

Lines 203-204: The analysis showed "no visible changes" in the 3D structure. What was the resolution of this analysis? Was it a simple visual inspection, or were quantitative metrics (e.g., changes in binding pocket volume, electrostatic surface potential, or protein stability) calculated? Could the mutations affect properties not visible in a static structure, like protein flexibility or interaction networks? Adding these data will contribute to the scientific content of the manuscript.

Lines 206-209: Regarding expression normalization: The study uses TPM for normalization. Was the "total mapped reads" used in the TPM denominator the total number of reads that mapped specifically to the TPNRBV genome, or was it the total number of reads in the entire sequencing library (including plant and other microbial reads)?

Lines 229-233: The phylogenetic trees for the different RNA segments were discordant (RNA1 vs. RNA2-4). The discussion states a close relationship to Hangzhou isolates but doesn't address this mixed signal. How do you interpret this conflicting phylogenetic history in the absence of more detectable recombination? Also, the discordant phylogenetic trees for RNA1 (groups with Fujian) versus RNA2-RNA4 (groups with Hangzhou) is a critical finding that is buried in the results and not adequately discussed. The discussion should explicitly address this and hypothesize about its cause, especially since recombination was weak. It would be better to mention these issues in the discussion.

Lines 245-248: The Iranian isolates share a pattern of conserved 5' terminal motifs with the Hangzhou isolate (absent in RNA2-RNA4, a specific variant in RNA1). Do you have data about the critical role of these motifs for viral replication? Does their shared, atypical structure provide stronger evidence for a direct evolutionary link than the nucleotide identity percentages? Enter this data for clarifying importance of the conserved terminal motifs.

Lines 250-253: The 110-nt insertion in RNA4 is a unique marker shared only by the Iranian and Hangzhou isolates. Does this insertion occur in a coding or non-coding region? What is its predicted functional impact, and does its presence override the phylogenetic signal from RNA1, making a Hangzhou origin more likely for the majority of the genome? This discussion would add the significance of the RNA4 insertion.

Lines 257-260: The study concludes the mapped reads were sufficient for full genome coverage. What was the minimum depth of coverage across the entire TPNRBV genome in each library, and were there any regions with consistently low coverage that might be prone to assembly errors?

Lines 267-271: The SNP data indicates a diverse quasispecies, while the phylogenetic trees show low variability. Given that the pooling strategy is the most likely cause, what specific follow-up experiment did you use to definitively resolve whether this diversity represents a true quasispecies within single plants or a mixture of very similar but distinct isolates across the plantation? Please clear this Quasispecies issue.

Lines 272-273: Were the 93 non-synonymous SNPs evenly distributed across the four segments of the TPNRBV genome (RNA1-RNA4), or were they concentrated in specific segments or genes (e.g., the highly expressed P22 gene)? What might a cluster of mutations in one particular gene suggest? Please add data regarding these questions to the results and discussion sections.

Lines 290-291: For the highly expressed P22 gene, what was the average read depth of coverage across its ORF? Was the read coverage uniform, or were there regions with unusually high or low coverage that might suggest post-transcriptional processing or assembly artifacts? Please add and discuss related results.

Lines 304-305: The statement that P22 has "no SNPs" should be nuanced. It would be more accurate to state that “no SNPs were found (above the 1% frequency threshold in this particular sample pool)”. This leaves open the possibility of lower-frequency variants or variation in other populations.

Lines 306-312: The Iranian isolates group with Fujian in the RNA1 tree but with Hangzhou in the RNA2-RNA4 trees. If RDP4 detected no recombination, what other evolutionary mechanisms could explain this apparent reassortment of genomic segments? Does this suggest the Iranian isolates could be a natural reassortant between two different Chinese TPNRBV lineages? Please enter these points in the discussion which will contribute to the article’s appeal and scientific clarity.

Lines 306-308: Given the high similarity to Chinese isolates, the mixed phylogeny, and the shared unique insertion, what is the most plausible hypothesis for the emergence of TPNRBV in Iran? Does the evidence point more strongly to a single introduction event from a population with mixed ancestry, or multiple independent introductions of different segments? It is recommended that this issue be discussed by including more data in addition to presenting the phylogeny trees.

Reviewer 3

The manuscript titled “First Complete Genome Sequences and Transcript Expression Profiling of Tea Plant Necrotic Ring Blotch Virus Isolates from Iran” by Esmaeilzadeh et al., report the first complete genome sequences of two Tea plant necrotic ring blotch virus (TPNRBV) isolates from Iran and analyze ORF expression (TPM) and within-host variation (SNPs) using pooled RNA-seq from 2021 and 2022. They show close relatedness to Chinese isolates, identify a 110-nt insertion in RNA4, and note high expression of P22. The work addresses a real gap in Iranian TPNRBV genomics and is relevant for tea pathology and regional surveillance.

Major strengths

• First complete TPNRBV genomes from Iran with segment-wise phylogenies (RNA1–RNA4).

• A coherent analysis package—assembly, expression, SNP calling, recombination, and phylogeny—from existing RNA-seq data.

• Clear practical motivation for disease monitoring and diagnostics in tea.

Points to improve (readability and rigor)

• Taxonomy/nomenclature. The species and common names are mixed (“Blunervirus camelliae (TPNRBV)”). Please standardize usage across the manuscript: species = Blunervirus camelliae; virus/common name = TPNRBV. Pick one convention and apply it consistently.

• Vector/transmission statements. The text mentions mechanical and seed transmission and hypothesized insect vectors (mites, aphids, leafhoppers, whiteflies). Please align these statements with current evidence for kitavirids/blunerviruses (e.g., Brevipalpus mites for related viruses) and cite primary sources. If evidence is lacking for TPNRBV, say so explicitly.

• Pooled samples and interpretation. Because samples were pooled by year, allele frequencies reflect mixtures of infections across plants, not clonal within-host dynamics. Likewise, TPM differences from pooled RNA-seq cannot be interpreted as within-host expression changes. Please reframe conclusions accordingly (no linkage inference; population-level, not within-host, signals).

• Phylogeny reporting. Specify the number of comparator isolates per segment and list all accessions (country/year/host) in the main text or a Supplementary Table. If you use “Clade I/II,” define the labels and the criteria for assignment.

• PCR verification details. Provide primer sequences (TPNRBV3-F/R, MP-F/R), exact annealing temperatures (rather than “respective temperatures”), and the counts of PCR-positive plants per pool. This will improve reproducibility.

• Results wording. The sentence “Low genetic variability between TPNRBV isolates based on the phylogenetic analysis indicates the absence of different isolates, and therefore the lack of a quasispecies structure.” over-interprets pooled data and conflicts with the reported SNPs.

Overall assessment. The manuscript is scientifically sound and generally well supported by relevant references. It integrates viral genome sequencing, expression profiling, SNP discovery, recombination screening, and segment-wise phylogeny for TPNRBV—highly relevant to tea pathology and surveillance—making this a potentially valuable contribution to the field. With careful revision along the points above, the work will be significantly stronger and its central claims clearer and it is highly recommended for publication after minor revision.

[NOTE: If reviewer comments were submitted as an attachment file, they will be attached to this email and accessible via the submission site. Please log into your account, locate the manuscript record, and check for the action link "View Attachments". If this link does not appear, there are no attachment files.]

To ensure your figures meet our technical requirements, please review our figure guidelines: https://journals.plos.org/plosone/s/figures

You may also use PLOS’s free figure tool, NAAS, to help you prepare publication quality figures: https://journals.plos.org/plosone/s/figures#loc-tools-for-figure-preparation.

NAAS will assess whether your figures meet our technical requirements by comparing each figure against our figure specifications.

# Editor report: Acceptance letter

**DOI:** 10.1371/journal.pone.0354634.r004

**Authors:** Murtada D. Naser

PONE-D-25-42547R1

PLOS One

Dear Dr. Koolivand,

I'm pleased to inform you that your manuscript has been deemed suitable for publication in PLOS One. Congratulations! Your manuscript is now being handed over to our production team.

At this stage, our production department will prepare your paper for publication. This includes ensuring the following:

* All references, tables, and figures are properly cited

* All relevant supporting information is included in the manuscript submission,

* There are no issues that prevent the paper from being properly typeset

You will receive further instructions from the production team, including instructions on how to review your proof when it is ready. Please keep in mind that we are working through a large volume of accepted articles, so please give us a few days to review your paper and let you know the next and final steps.

Lastly, if your institution or institutions have a press office, please let them know about your upcoming paper now to help maximize its impact. If they'll be preparing press materials, please inform our press team within the next 48 hours. Your manuscript will remain under strict press embargo until 2 pm Eastern Time on the date of publication. For more information, please contact onepress@plos.org.

You will receive an invoice from PLOS for your publication fee after your manuscript has reached the completed accept phase. If you receive an email requesting payment before acceptance or for any other service, this may be a phishing scheme. Learn how to identify phishing emails and protect your accounts at https://explore.plos.org/phishing.

If we can help with anything else, please email us at customercare@plos.org.

Thank you for submitting your work to PLOS ONE and supporting open access.

Kind regards,

PLOS ONE Editorial Office Staff

on behalf of

Dr. Murtada D. Naser

Academic Editor

PLOS One
